# A Complex Interplay of Anionic Phospholipid Binding Regulates 3′-Phosphoinositide-Dependent-Kinase-1 Homodimer Activation

**DOI:** 10.1038/s41598-019-50742-8

**Published:** 2019-10-10

**Authors:** Gloria de las Heras-Martínez, Véronique Calleja, Remy Bailly, Jean Dessolin, Banafshé Larijani, Jose Requejo-Isidro

**Affiliations:** 1 Instituto Biofisika (CSIC, UPV/EHU), 48490 Leioa, Spain; 2 Protein Phosphorylation Laboratory, The Francis Crick Institute, 1 Midland Road, NW1 1AT London, UK; 3 Institute of Chemistry & Biology of Membranes & Nanoobjects (UMR 5248 CBMN) CNRS – Université de Bordeaux - Bordeaux INP All. Geoffroy Saint-Hilaire, 33600 Pessac, France; 4 Cell Biophysics Laboratory, Ikerbasque Basque Foundation for Science, Instituto Biofisika (CSIC, UPV/EHU) & Research Centre for Experimental Marine Biology and Biotechnology (PiE), University of the Basque Country (UPV/EHU), Leioa, 48940 Spain; 5 Centre for Therapeutic Innovation (CTI-Bath); Cell Biophysics Laboratory Department of Pharmacy & Pharmacology University, Bath, Claverton Down, Bath, BA2 7AY United Kingdom; 6 Centro Nacional de Biotecnología (CSIC), Darwin, 3, E28049 Madrid, Spain; 7 Unidad de Nanobiotecnología, CNB-CSIC-IMDEA Nanociencia Associated Unit, 28049 Madrid, Spain

**Keywords:** Molecular conformation, Lipid signalling

## Abstract

3′-Phosphoinositide-dependent-Kinase-1 (PDK1) is a master regulator whereby its PI3-kinase-dependent dysregulation in human pathologies is well documented. Understanding the direct role for PtdIns(3,4,5)P_3_ and other anionic phospholipids in the regulation of PDK1 conformational dynamics and its downstream activation remains incomplete. Using advanced quantitative-time-resolved imaging (Fluorescence Lifetime Imaging and Fluorescence Correlation Spectroscopy) and molecular modelling, we show an interplay of antagonistic binding effects of PtdIns(3,4,5)P_3_ and other anionic phospholipids, regulating activated PDK1 homodimers. We demonstrate that phosphatidylserine maintains PDK1 in an inactive conformation. The dysregulation of the PI3K pathway affects the spatio-temporal and conformational dynamics of PDK1 and the activation of its downstream substrates. We have established a new anionic-phospholipid-dependent model for PDK1 regulation, depicting the conformational dynamics of multiple homodimer states. We show that the dysregulation of the PI3K pathway perturbs equilibrium between the PDK1 homodimer conformations. Our findings provide a role for the PtdSer binding site and its previously unrewarding role in PDK1 downregulation, suggesting a possible therapeutic strategy where the constitutively active dimer conformer of PDK1 may be rendered inactive by small molecules that drive it to its PtdSer-bound conformer.

## Introduction

3′-Phosphoinositide-dependent-Kinase-1 (PDK1) propagates extracellular signals downstream of Phosphoinositide 3-kinase (PI3K) through the phosphorylation of a plethora of AGC kinases involved in growth, survival and cell motility^1,2^. PDK1 has many kinase-dependent and independent functions, and fulfils the criteria of being a master regulator of proliferative signalling. The physiological evidence is clear as alteration in PDK1 expression or activity have been found many types of cancer^3^. PDK1 can associate with the plasma membrane (PM) by interaction with phosphatidylinositol (3,4,5) trisphosphate (PtdIns(3,4,5)P_3_), phosphatidylinositol (4,5) bisphosphate (PtdIns(4,5)P_2_) and phosphatidylserine (PtdSer) via its pleckstrin homology (PH) domain^4–6^, and through other adaptor proteins such as Grb14^7^, Grp78^8^ and Freud-1/Aki1^9^. Despite being mainly localised in the cytoplasm and at the PM, pools of PDK1 have been detected in the nucleoplasm where many of its substrates also reside^10–13^. The high-affinity specific interaction of PDK1 with PtdIns(3,4,5)P_3_ through its K465 residue on its PH domain has been well established^5,14,15^. However, reports on whether PtdIns(3,4,5)P_3_ is solely responsible for PDK1 recruitment to the PM are contradictory^4,6,16^, suggesting a complex interplay between PDK1 binding to phosphoinositides and other anionic phospholipids. PtdSer is the predominant anionic species and is present at higher concentrations than the phosphoinositides at the inner leaflet of the PM^17^. PtdSer has been reported to be involved in the membrane localisation of PDK1 through its basic residues R466 and K467^6^ but the role of this interaction has not yet been fully elucidated. Besides, it has been shown that PDK1 is constitutively active since it is phosphorylated on its activation loop at S241 and is active *in vitro*^18^. However, it was also shown *in situ* that PDK1 itself could be regulated upon stimulation^19,20^. More recently, PtdIns(3,4,5)P_3_ binding to PDK1 in live cells was shown to elicit the formation of PDK1 homodimers^13,19^ and to trigger the autophosphorylation of the PDK1 PH domain residue 513. This, was suggested to be due to the disruption of an autoinhibitory PDK1 homodimer conformer^13,21,22^, although this mechanism still remains to be defined.

These findings pointed towards an elaborate fine-tuning of PDK1 conformational dynamics upon stimulation in cells that involve the interaction with negatively charged lipids such as PtdIns(3,4,5)P_3_, PtdIns(4,5)P_2_ and PtdSer, autophosphorylation of T513 and homodimerisation. However, our understanding of the interplay between these events and the associated regulation that enables the phosphorylation of PDK1 substrates remains largely unknown. Addressing these issues has been hampered by the lack of suitable *in situ* quantitative methods for interrogating the spatio-temporal sequence of protein-lipid interactions. For this reason, we recently developed a precise and well-resolved quantitative imaging method based on FRET-FLIM (Förster Resonance Energy Transfer - Fluorescence Lifetime Imaging) that overcomes these technical obstacles^23^. In this work, we have been able to monitor *in situ* the anionic phospholipid-mediated regulation of PDK1 homodimerisation and localisation leading to PDK1 activation, and how this is linked to T513 autophosphorylation in cells with a normal or a dysregulated PI3K pathway. Using the methodology we have recently developed^23^, we unravelled three major mechanisms for PDK1 regulation. Firstly, that PDK1 homodimerises in an activated conformation phosphorylated on T513 and is capable of activating downstream substrates like PKB/Akt and SGK1. Secondly, that this homodimer formation is triggered by two opposite mechanisms: (i) The binding to PtdIns(3,4,5)P_3_ upon growth factor stimulation, and (ii) the loss of PM binding to other anionic phospholipids. This suggested a competitive regulatory mechanism involving PtdIns(3,4,5)P_3_ and other anionic phospholipids having opposing effects on PDK1 activation. Thirdly, we have demonstrated that PDK1 activation through recruitment to the PM is not only dependent on its PH domain but its kinase domain plays a prominent role in this process. The role of the kinase domain interaction is of particular importance in cells with a dysregulated PI3K pathway and an aberrant regulation of the PtdIns(3,4,5)P_3_ levels, leading to an abnormal PDK1 activation.

## Results

### Characterisation of anionic lipids binding to PDK1 PH domain *in vitro*

**Figure 1:**
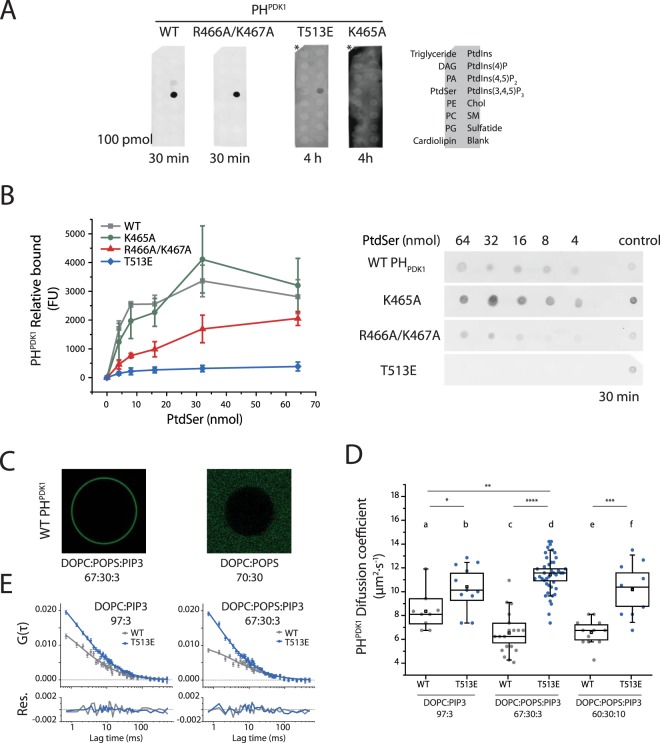
Characterisation of anionic lipids binding to PDK1 PH domain *in vitro* (**A)**. Lipid protein-overlay assays showing the binding of WT-PH^PDK1^ and mutants to a selection of lipids spotted on nitrocellulose. **(B)** PH^PDK1^ wild type and mutants’ affinity for PtdSer. **(C)** Confocal images of WT-PH^PDK1^ association to GUVs of different compositions. **(D)** WT-PH^PDK1^ and mutant T513E-PH^PDK1^ diffusion on GUVs of different composition. **(E)** Representative autocorrelation curves from sFCS experiments, each corresponds to one GUV. N > 8. Box: 2xSEM (Standard Error of the Mean); Whiskers: 80% population. Mann-Whitney test *p < 0.05.

The nature of PDK1 interactions and regulation by anionic phospholipids at the PM remains a subject of discussion. To understand the mechanism of interplay of the anionic phospholipids associations with PDK1, we characterised their *in vitro* interactions using fluorescently tagged PH domains of PDK1 (PH^PDK1^). PH^PDK1^ wild type (WT) and mutants binding to specific lipid species was tested using a protein-lipid overlay assay, while the nature of their association to the bilayer was studied through their diffusional behaviour using single-focus scanning Fluorescence Correlation Spectroscopy (sFCS)^24,25^ (Figs 1 and S1). The positively charged amino acid site 465, previously associated with PtdIns(3,4,5)P_3_, and 466/467, associated with PtdIns(4,5)P_2_ and PtdSer binding^5,6^, were mutated to the neutral residues alanine (K465A and R466A/K467A) (Figs S2A and S3). As a preliminary indication we utilised a protein-lipid overlay assay, to determine the PH^PDK1^ affinity for PtdIns(3,4,5)P_3_, PtdIns(4,5)P_2_ and PtdSer. Although this assay does not reflect the physiological behaviour of the PH domains towards the anionic lipids, it is still indicative of their *in vitro* binding affinity. We showed that WT-PH^PDK1^ bound PtdIns(3,4,5)P_3_ with a high affinity and PtdIns(4,5)P_2_ to a lesser extent (Fig. 1A). In line with previous reports, detectable interactions with PtdIns(3,4,5)P_3_ or PtdIns(4,5)P_2_ were not observed with the K465A-PH^PDK1^ mutant even during extended incubations^5^. The R466A/K467A-PH^PDK1^ mutant, however, selectively lost its binding to PtdIns(4,5)P_2_ but not to PtdIns(3,4,5)P_3_.

**Figure 2:**
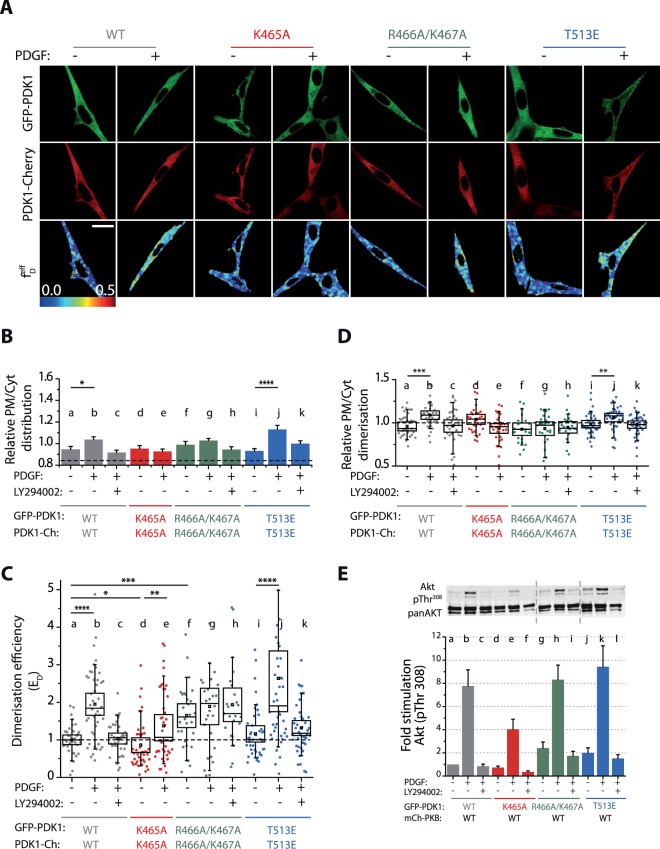
Opposing effects of anionic phospholipids regulate the formation of active PDK1 homodimers. All experiments were performed under resting conditions (−), stimulation of the PI3K pathway with PDGF (+) or treated with the PI3K inhibitor (LY294002) prior to stimulation. NIH3T3 cells were cotransfected with GFP-PDK1 and PDK1-mCherry. **(A)** Representative intensity and FRET-FLIM *f*_*D*_ images. **(B)** Relative distribution of PDK1 at the PM and the cytoplasm of NIH3T3 cells. The dashed line indicates the reference level for a control cytoplasmic protein (Table 1). **(C)** Quantification of the homodimerisation efficiency of PDK1 wild type and mutants. *E*_D_ calculation is based on FRET-FLIM measurements on a single-cell level, as illustrated in **(A)**. **(D)** Localisation of the PDK1 homodimers in NIH3T3 cells. The graph shows the ratio of PDK1 dimers at the PM and the cytoplasm based on the segmentation of the *f*_*D*_ images shown in (**A**). **(E)** Phosphorylation of Akt/PKB at T308 in NIH3T3 cells. The upper panel shows a representative western blot of phosphorylated Akt/PKB on T308 and of total Akt/PKB (pan AKT). The bars show the ratio of T308 phosphorylation over total protein. The data were normalised to the condition basal wild type PDK1. Scale bar: 20 µm. **(B)** N > 30. Error bars: SEM. **(C,D)** N > 30. Box: 2xSEM (95.4% confidence); Whiskers: 80% population. Mann-Whitney test *p < 0.05. **(B-E)** Three independent experiments.

**Figure 3:**
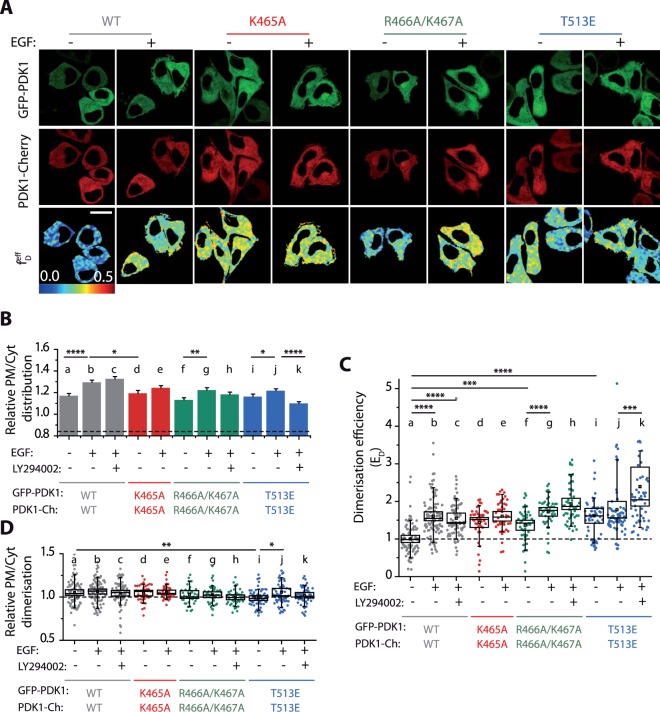
In PI3K-dysregulated cells, PDK1 homodimerisation and localisation is independent of PtdIns(3,4,5)P_3_ levels. All experiments were performed under resting conditions (−), stimulation of the PI3K pathway with EGF (+) or treated with the PI3K inhibitor (LY294002) prior to stimulation. SKBR3 cells were cotransfected with GFP-PDK1 and PDK1-mCherry. **(A)** Representative intensity and FRET-FLIM *f*_*D*_ images. **(B)** Relative distribution of full-length PDK1 at the PM and the cytoplasm of SKBR3 cells. The dashed line indicates the reference level for a control cytoplasmic protein (Table 1). **(C)** Quantification of the homodimerisation efficiency of full-length WT PDK1 and its mutants. *E*_D_ calculation is based on FRET-FLIM measurements on a single-cell level, as illustrated in **(A)**. **(D)** Localisation of the PDK1 homodimers in SKBR3 cells. The graph shows the ratio of PDK1 dimers at the PM and the cytoplasm based on the segmentation of the *f*_*D*_ images shown in **(A)**. Scale is 20 µm. N > 30. Error bars: SEM. **(C,D)** N > 30. Box: 2xSEM; Whiskers: 80% population. Mann-Whitney test *p < 0.05. Three independent experiments.

**Figure 4:**
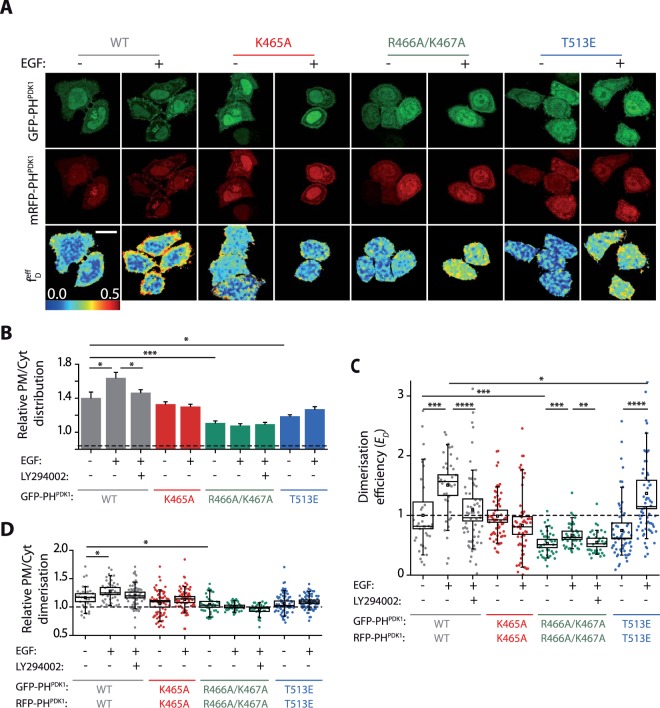
The homodimerisation and localisation of PDK1 PH domain follows PtdIns(3,4,5)P_3_ levels, contrary to full-length PDK1. All experiments were performed under resting conditions (−), stimulation of the PI3K pathway with EGF (+) or treated with the PI3K inhibitor (LY294002) prior to stimulation. SKBR3 cells were cotransfected with GFP- and mRFP- tagged PH^PDK1^. **(A)** Representative intensity and FRET-FLIM *f*_*D*_ images. **(B)** Relative distribution of PH^PDK1^ at the PM and the cytoplasm of SKBR3 cellx. The dashed line indicates the reference level for a control cytoplasmic protein (Table 1). **(C)** Quantification of the homodimerisation efficiency of WT PH^PDK1^ and its mutants. **(D)** Localisation of the PH^PDK1^ homodimers in SKBR3 cells. The graph shows the ratio of PH^PDK1^ dimers at the PM and the cytoplasm based on the segmentation of the *f*_*D*_ images shown in **(A)**. Scale is 20 µm. N > 30. Error bars: SEM. **(C,D)** N > 30. Box: 2xSEM; Whiskers: 80% population. Mann-Whitney test *p < 0.05. Three independent experiments.

Since the residues R466/K467 were previously proposed to be necessary for PDK1 interaction with PtdSer^6^ we investigated the binding properties of the different mutants with this phospholipid. PtdSer binding to PH^PDK1^ was only detected after a 40-fold increase of the amount of lipid (from 100 pmol in Fig. 1A to 4 nmol in Fig. 1B), demonstrating that the *in vitro* PH^PDK1^ affinity for PtdSer was significantly lower than PtdIns(3,4,5)P_3_. The mutation K465A did not affect binding to PtdSer, while the mutation R466A/K467A showed a four-fold reduction in affinity. These data suggested that despite the close proximity of these three residues (K465, R466A and K467A) on the PDK1 PH domain, their different binding properties enabled to discriminate the effect of PtdIns(3,4,5)P_3_ from the other anionic phospholipids.

We next determined whether post-translational modifications of the PH domain could also regulate PDK1 binding to the PM by affecting its association with anionic phospholipids. We and others had previously shown that PDK1 activation was dependent on the autophosphorylation of the PH domain residue T513 upon PM translocation^13,21,24^. Therefore, we examined whether T513 phosphorylation affected the binding of PH^PDK1^ to anionic phospholipids. To this aim we prepared a PH^PDK1^ mutant that mimicked PH^PDK1^ phosphorylation on T513 (T513E-PH^PDK1^)^24^. Figure 1A shows that even though T513E-PH^PDK1^ retained some ability to bind PtdIns(3,4,5)P_3_ it required an extended incubation time to be detected, reflecting a lower affinity for this lipid compared to the wild type species. In addition, T513E-PH^PDK1^ did not bind PtdSer even at the highest concentrations (Fig. 1B). These experiments suggested that the autophosphorylation of the PDK1 PH domain residue 513 upon stimulation could trigger a loss of affinity of the activated PDK1 for the PM.

To further understand the nature of the PDK1 – anionic lipid interaction we studied PH^PDK1^ association and diffusion on giant unilamellar vesicles (GUV) composed of DOPC (1,2-di-(9Z-octadecenoyl)-sn-glycero-3-phosphocholine), PtdIns(3,4,5)P_3_ (PIP3) and PtdSer (1-hexadecanoyl-2-(9Z-octadecenoyl)-sn-glycero-3-phospho-L-serine, POPS) mixed at varying proportions using sFCS (Methods and Fig. S1). Diffusion of a protein on a lipid bilayer depends largely on the lipid-protein electrostatic interactions. A protein tightly bound to an individual lipid through a stereospecific, lipid-specific, interaction diffuses at the same rate as the lipid irrespective of the surface potential of the vesicle^26^. However, a protein associated to the vesicle through non-specific electrostatic interactions slides on the GUV surface faster than individual lipids in the mixture, since its diffusion is, in this case, subject only to the charge-driven attraction between the protein positive patches and the GUV negatively charged surface. Consequently, the diffusion coefficient of the protein is only effected by modulation of the membrane surface potential in the latter case, but not in the former^26^. In this sense the diffusion of a protein on a model membrane does not relate directly to protein function, but provides information on protein-membrane interaction, which is later correlated to its function.

Confocal microscopy of the GUVs showed that wild type PH^PDK1^ readily bound the outside layer of a GUV containing only 3% PtdIns(3,4,5)P_3_ (DOPC:POPS:PIP3 67:30:3), but binding was not detected when PtdIns(3,4,5)P_3_ was absent from the mixture (DOPC:POPS 70:30 GUVs) (Fig. 1C). This behaviour reflected the results obtained from the protein-lipid overlay assay.

The diffusion coefficient of WT-PH^PDK1^ on DOPC:PIP3 (97:3) vesicles was 8.3 ± 0.5 µm^2^ s^−1^ (Fig. 1D) which is highly consistent with the reported diffusion coefficient of individual lipids in fluid mixtures^27^, suggesting a lipid-specific association of WT-PH^PDK1^ to PtdIns(3,4,5)P_3_ that was later confirmed (below). Given the weak binding of PH^PDK1^ to PtdSer compared to PtdIns(3,4,5)P_3_ (Fig. 1B), and that association of PH^PDK1^ to DOPC:POPS (70:30) GUVs was not observed, we did not anticipate any effect of PtdSer in the diffusion of PH^PDK1^. We found, however, that PH^PDK1^ diffused faster on PtdIns(3,4,5)P_3_-containing GUVs that lacked PtdSer (DOPC:PIP3 97:3, diffusion coefficient 8.3 ± 0.5 µm^2^ s^−1^) than on GUVS that contained POPS and the same mole fraction of PtdIns(3,4,5)P_3_, (DOPC:POPS:PIP3 67:30:3, diffusion coefficient 6.5 ± 0.4 µm^2^ s^−1^, Fig. 1D). This indicated that PtdSer was able to bind PH^PDK1^ only in combination with PtdIns(3,4,5)P_3_ binding. This dual association maybe due to either the binding of the 2 anionic phospholipids to the same PH domain or to the formation of a dimeric conformation where each lipid would bind one PH domain (sFCS alone would not be able to distinguish between a monomeric PH conformer versus a dimeric PH conformer). We did not observe any change in the diffusion of PH^PDK1^ on DOPC:POPS:PIP3 GUVs when the mole fraction of the polyvalent, highly negative PtdIns(3,4,5)P_3_ was increased from 3% to 10% (6.5 ± 0.4 µm^2^ s^−1^ and 6.6 ± 0.3 µm^2^ s^−1^), confirming that PH^PDK1^ bound PtdIns(3,4,5)P_3_ in a lipid-specific manner, rather than by a non-specific, charge-driven, interaction. Finally, the phosphomimetic T513E-PH^PDK1^ mutant diffused in a similar manner in GUVs with different lipid compositions and showed faster diffusion than WT-PH^PDK1^ in DOPC:PIP3 GUVs (Fig. 1Db,d,f). Since the affinity of T513E-PH^PDK1^ for PtdIns(3,4,5)P_3_ was very low, and negligible for PtdSer, this behaviour indicated the predominance of non-specific, electrostatically driven attraction of T513E PH^PDK1^ positive patches to the negatively charged vesicle surface.

### Opposing effects of anionic phospholipids regulate the formation of activated PDK1 homodimers

Having established the *in vitro* binding properties of the different PDK1 phospholipids mutants and determined the effect of PtdSer on the binding of PDK1 PH domain to PtdIns(3,4,5)P_3_, we examined the interplay of these lipids in the regulation of full-length PDK1 in cells. To this end, we analysed the activation potential of wild type PDK1 and relevant PDK1 mutants through the study of their localisation and conformational dynamics. An activated PDK1 conformer refers, in this context, to the molecular conformation that enables the phosphorylation of its downstream targets.

We had previously reported PDK1 homodimerisation in response to growth factor stimulation^13^. However, in our previous work, the interacting population of PDK1 had not been directly determined. In this investigation we directly quantified the interacting population of full-length protein PDK1 homodimers *in situ* using quantitative FRET-FLIM (Supplementary Methods and Fig. S4). FRET was detected by FLIM as the energy transfer from ectopically expressed eGFP-myc-PDK1 (donor) to HA-PDK1-mCherry (acceptor) (Fig. S2B). Here, based on fluorescence lifetime analysis we determined the effective fraction of donor undergoing FRET, ${f}_{D}^{eff}$, for every cell (Fig. S4A,B) and defined the Dimerisation Efficiency (*E*_*D*_). As opposed to the FRET efficiency, which is dependent on the relative abundance of interacting partners, *E*_D_ directly accounts for cell-to-cell variations in donor and acceptor concentration (Fig. S4C–E). With this methodology, *E*_D_ allows comparison between cells, while ${f}_{D}^{eff}$, which can be determined for every pixel in the cell, allows comparison between regions in the same cell.

Due to the limited resolution of optical microscopy, protein translocation to the PM cannot be reliably assessed from visual inspection of confocal images, especially in the case of weak translocation. For this reason, to obtain a quantitative measurement of the recruitment of total PDK1 and dimeric PDK1 at the PM in response to growth factor stimulation we utilised image segmentation methods on intensity and FRET-FLIM images to determine the spatial localisation of PDK1 as well as the localisation of PDK1 homodimers, respectively (Figs S4F and S5). We compared the ratio of PDK1 fluorescent intensity at the PM to the intensity at the cytoplasm (PM/Cyt) to that of a cytoplasm targeted construct (freely diffusing mCherry-6Gly-EGFP) and to a membrane-targeted construct (a Lyn N-terminal sequence fused to a FKBP domain, Lyn_11_-FKBP-FKBP-mRFP^28^). Finally, segmentation of the FRET-FLIM images allowed computation of the ${f}_{D}^{eff}$ ratio at the PM and cytoplasm, which is a measurement of the localisation of the molecules undergoing FRET. As a reference for the relative PM/Cyt distribution of GFP-PDK1 – mCherry-PDK1 dimers we used the inherently FRETting mCherry-6Gly-EGFPT construct^23^. All these reference translocation levels are indicated in Table 1.

**Table 1:** Translocation reference levels for cytoplasm- and membrane-targeted fluorescent control constructs measured through the EGFP, mCherry, and FRET-FLIM imaging detectors (supporting methods) at different pixel sizes.

Pixel size (nm)	Cytoplasm targeted			Membrane targeted
	Free-diffusing mCherry-6Gly-EGFP			Lyn_11_-FKBP_2_-mRFP
	EGFP *PM/Cyt intensity*	mCherry *PM/Cyt intensity*	FRET-FLIM ${f}_{D}^{eff}$ *PM/Cyt*	mCherry channel *PM/Cyt intensity*
275	0.84 ± 0.01	0.85 ± 0.01	1.04 ± 0.01	2.2 ± 0.1
68	0.99 ± 0.01	0.98 ± 0.01	—	2.7 ± 0.1

**Figure 5:**
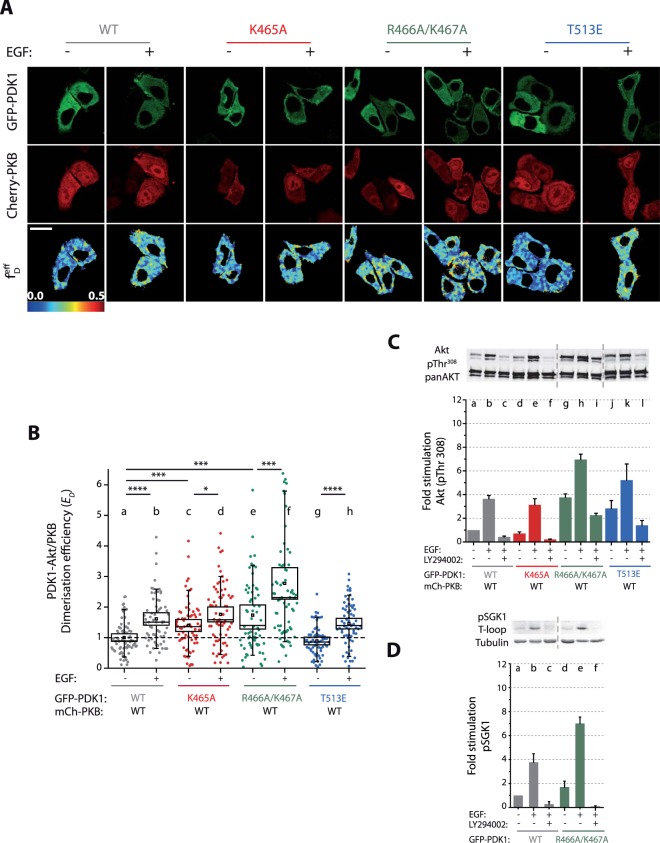
Akt/PKB heterodimerisation with PDK1 and downstream activation correlates with the level of PDK1 homodimerisation in SKBR3 cells. (**A**) Representative intensity and FRET-FLIM images of SKBR3 cells co-transfected with mCherry-Akt/PKB and GFP-PDK1. (**B**) The efficiency of mCherry-Akt/PKB and GFP-PDK1 interaction determined by FRET-FLIM in SKBR3 cells increases upon growth-factor stimulation and is highest for R466A/K467A PDK1 mutant. (**C**) Phosphorylation of Akt/PKB at T308 correlates well with the efficiency of Akt/PKB and PDK1 heterodimerisation. (**D**) Phosphorylation of endogenous SGK1 at its T-loop by PDK1 is potentiated by the mutation R466A/K467A. (**A**) Scale bar: 20 µm. (**B**) N > 30. Box: 2xSEM; Whiskers: 80% population. Mann-Whitney test *p < 0.05. Three independent experiments. (**C**–**D**) n = 3. Error bars: SEM.

275 nm pixel-size images were acquired for FRET experiments (Figs 2–5), while 68 nm pixel-size images were acquired for the quantification of PtdIns(3,4,5)P_3_ levels (Fig. S6). The difference between PM/Cyt intensity values for 275 nm and 68 nm pixel size is due to the lower resolution of the former. ${f}_{D}^{eff}$ PM/Cyt is the PM to cytoplasm ${f}_{D}^{eff}$ ratio, which quantifies the relative distribution of FRETting pairs. The uncertainty is indicated as SEM for at least N > 20.

Initially, we analysed GFP-PDK1 and PDK1-mCherry recruitment at the PM in NIH3T3 cells irrespective of their dimerisation status by segmentation of the intensity images (Fig. 2A). As compared to the membrane- and cytoplasm- targeted reference controls (PM/Cyt intensity 2.2 ± 0.1 and 0.84 ± 0.1 respectively), intensity segmentation indicated a predominantly cytoplasmic distribution of GFP-PDK1 and PDK1-mCherry in resting NIH3T3 cells (Fig. 2B). PDK1 translocated weakly to the PM following an increase of PtdIns(3,4,5)P_3_ upon platelet-derived growth factor (PDGF) stimulation, and this was prevented by the PtdIns(3,4,5)P_3_-binding site mutation K465A. In all cases, PDK1 recruitment to the NIH3T3 PM was compromised when PI3K was inhibited by LY294002 prior to stimulation. Nevertheless, despite the weak affinity of the isolated PH domain mutant T513E for PtdIns(3,4,5)P_3_ and other phospholipids *in vitro* (Fig. 1), full-length PDK1-T513E recruited to the PM upon PDGF stimulation. This result suggested a potential role for PDK1 kinase domain, either directly binding to the PM or by an allosteric effect on the PH domain affinity for the PM.

The mutation of the PDK1 PH domain residues R466A/K467A had been previously described to impair the binding of PDK1 to PtdSer and the localisation at the PM of NIH3T3 cells^6^. Our *in vitro* experiments showed that, in addition to PtdSer, the mutation also precluded the binding to PtdIns(4,5)P_2_ (Fig. 1A). Nonetheless the overall localisation of full-length GFP-PDK1 and PDK1-mCherry in NIH3T3 cells was not affected by the R466A/K467A mutation (Fig. 2Bf–h).

Next, we monitored the homodimerisation of PDK1 by acquiring, *in situ*, full-length GFP-PDK1 and PDK1-mCherry wild type and mutants homodimerisation by FRET-FLIM. We observed that dimer formation (Fig. 2C) and dimer localisation (Fig. 2D) were not systematically following the localisation pattern determined for the total amount of protein, described above (observations for total protein, based on intensity measurements, did not take into account whether the protein was in a dimer form). The *E*_D_ values for WT PDK1 showed an increase of PDK1 homodimerisation in response to growth factor (PDGF) stimulation, while it remained at basal level when PI3K was inhibited by LY294002 prior to stimulation. Consistent with the latter, the loss of PtdIns(3,4,5)P_3_ binding (K465A) precluded the strong dimerisation of PDK1 upon PDGF stimulation. A small increase in *E*_*D*_ was still detected possibly due to unspecific electrostatic interactions (Fig. 2Cd-e).

Mimicking PDK1 phosphorylation with the PH domain mutation T513E had been shown to increase PDK1 activity^13,24^. Additionally, residue 513 phosphorylation is known to relieve a PDK1 autoinhibitory conformation^21^. This led to the hypothesis that T513 phosphorylation induced a disruption of PDK1 autoinhibited homodimers resulting in the formation of active PDK1 monomer^13,21^. However, by determining the E_D_ we illustrate here that T513E mutation did not prevent PDK1 homodimerisation (Fig. 2Ci–k) and that the dimer regulation was similar to the WT-PDK1. This demonstrated that the activation of PDK1 by phosphorylation at 513 did not induce PDK1 monomers but resulted in an enhancement of PDK1 homodimer population.

The loss of PtdSer and PtdIns(4,5)P_2_ binding through the R466A/K467A mutation did not prevent PDK1 homodimerisation (Fig. 2Cf–h). On the contrary, the population of R466A/K467A-PDK1 dimers was significantly higher than the WT-PDK1 in basal conditions and did not change upon either growth factor stimulation or PI3K inhibition. Since the constitutive formation of the R466A/K467A-PDK1 homodimers was independent of the modulation of PI3K, these data suggested that R466A/K467A-PDK1 homodimer formation, unlike WT-PDK1, occurred through a mechanism independent of its binding to PtdIns(3,4,5)P_3_ and other anionic phospholipids.

To understand the mechanisms of PDK1 homodimer formation, we specifically analysed their subcellular localisation. Image segmentation of the FRET-FLIM images allowed the PM/Cyt ratio of GFP-PDK1 and PDK1-mCherry FRET fraction (${f}_{D}^{eff}$) to be computed, revealing differences between the localisation of PDK1 homodimer and total PDK1 (irrespective of its homodimerisation status). Segmentation of the FRET-FLIM images (Fig. 2D) demonstrated that WT-PDK1 dimers were recruited to the PM in NIH3T3 upon the increase of PtdIns(3,4,5)P_3_ levels. The localisation of the PDK1 mutants that did not bind PtdIns(3,4,5)P_3_ via their PH domain (K465A) revealed that they were still retained at the PM in basal conditions, indicating that the localisation of the PDK1 homodimers was due to PM interactions involving other factors than the binding of the PH domain to PtdIns(3,4,5)P_3_. The localisation of the T513E mutant followed the pattern of the wild type protein, suggesting that the mechanisms involved in the formation of the WT and T513E-PDK1 homodimers were similar. The R466A/K467A-PDK1 dimers did not localise to the PM even after growth factor stimulation but they predominantly localised in the cytoplasm. Therefore, the R466A/K467A-PDK1 mutant behaviour suggests a different mechanism for the formation of PDK1 homodimers.

Our results demonstrated that the increased formation of PDK1 homodimers was not only the result of growth factor stimulation, but could also occur in a constitutive manner due to mutation. We therefore determined the activation status of WT-PDK1 and its mutants. Our results had also shown that the activating mutant T513E-PDK1 was also a homodimer, raising the possibility that the homodimer conformer of PDK1 would be activated and capable of phosphorylating downstream targets. Therefore, we monitored the effect of the mutants on the phosphorylation of Akt/PKB, a PDK1 substrate. Figure 2E shows the direct activation of Akt/PKB by PDK1 reported by the phosphorylation of the T308 residue in Akt/PKB’s activation loop. Phosphorylation of Akt/PKB by WT-PDK1 increased upon PDGF stimulation. This increase correlated with the augmentation of WT-PDK1 homodimers and their localisation at the PM (Fig. 2Cb,Db). PDK1 PtdIns(3,4,5)P_3_-binding defective mutant K465A moderated T308 phosphorylation increase upon stimulation, but did not abolish it completely. This also correlated with K465A-PDK1 basal localisation at the PM and residual formation of these mutant homodimers upon stimulation (Fig. 2Bd,Cd).

Both the activating T513E-PDK1 and the PM localisation-defective R466A/K467A-PDK1 triggered an increase in T308 phosphorylation upon stimulation that was similar to WT-PDK1 (Fig. 2E). This also correlated to WT-PDK1, T513E and R466A/K467A elevated homodimer formation, despite their different localisation patterns (Fig. 2C,D). It is important to note that, irrespective of PDK1 homodimerisation status, Akt/PKB requires prior binding to PtdIns(3,4,5)P_3_ before being phosphorylated by PDK1 at T308^10^. Therefore, in resting conditions or after PI3K inhibition, Akt/PKB phosphorylation was reduced despite the increased levels of PDK1 homodimers (Fig. 2Eg,i,Cf,h). Both T513E and R466A/K467A mutations induced a slightly higher basal phosphorylation of Akt/PKB compared to WT, reinforcing the notion that PDK1 activation followed closely its homodimerisation status.

Taken together, these data suggested that growth factor stimulation was promoting the formation of active PDK1 homodimers through binding to PtdIns(3,4,5)P_3_ at the PM, although the homodimerisation and localisation patterns of PDK1 mutants hinted to the existence of a more nuanced regulation mechanism. We suggest an alternative mechanism whereby the formation of active PDK1 homodimers may bypass binding to PtdIns(3,4,5)P_3_. We propose that PtdIns(3,4,5)P_3_ and other anionic phospholipids concurred to localise PDK1 at the PM but their differential binding triggered opposite effects on PDK1 regulation. PtdIns(3,4,5)P_3_-binding induced a conformation that was conducive to PDK1 and downstream substrates activation, whereas the binding to anionic phospholipids other than PtdIns(3,4,5)P_3_ maintained PDK1 in an inactive conformation at the PM.

To understand the dynamics of anionic phospholipids associated to the mechanism of PDK1 regulation, we took advantage of a cell line with a dysregulated PI3 kinase and an altered phosphoinositide composition. SKBR3 cells are a breast cancer cell line with overexpressed levels of HER2 inducing anomalies in the PI3 kinase pathway. First we investigated the modulation of PtdIns(3,4,5)P_3_ by growth factor stimulation and PI3K inhibition and the PtdSer levels in these cells. This, in turn, was compared to how these anionic phospholipids affected the formation of dimers of PDK1 and its mutants in SKBR3.

### Determination of the relative *in situ* levels of PtdIns(3,4,5)P_3_ by GRP1 PH domain

To test how the aberrant behaviour of the HER2 growth factor receptor pathway in SKBR3 cells affected the levels of PtdIns(3,4,5)P_3_, as compared to NIH3T3 cells, we used an *in situ* intensity segmentation method to quantify PtdIns(3,4,5)P_3_ levels (Fig. S6). In these experiments, we specifically chose a PH domain probe with a relatively low affinity for PtdIns(3,4,5)P_3_ detection (GFP-GRP1^PH^)^29^. The lower affinity of this probe (*K*_D_ ≈ 50 nM^30^), compared to PH^PDK1^, (*K*_D_ ≈ 0.2 nM^31^) reduced the cytoplasmic sequestration of PtdIns(3,4,5)P_3_ and thus provided a more accurate representation of endogenous levels of PtdIns(3,4,5)P_3_ at the PM. PtdIns(3,4,5)P_3_ levels at the PM upon stimulation of the PI3K pathway were higher in SKBR3 cells than in NIH3T3 cells (23% and 8% increase relative to the basal state, respectively) (Fig. S6C,D). PI3K inhibition (LY294002) prior to stimulation resulted in the decrease of PtdIns(3,4,5)P_3_ levels at the PM below the basal levels observed in NIH3T3 cells, while they remained at the basal level in SKBR3 cells. The GFP-GRP1^PH^ probe followed the same behaviour in starved and non-starved SKBR3 cells (Fig. S6E). The control binding-defective GRP1 PH domain mutants did not bind to PtdIns(3,4,5)P_3_. These data showed that, unlike NIH3T3 cells, the basal level of PtdIns(3,4,5)P_3_ in SKBR3 was not affected by PI3K inhibitor (LY294002). This suggested either an additional PI3K independent source of PtdIns(3,4,5)P_3_ or that elevated PtdIns(3,4,5)P_3_ was a consequence of enhanced receptor tyrosine kinase activity in SKBR3 cells and the residual uninhibited PI3K. In summary, the regulation of the PtdIns(3,4,5)P_3_ levels was altered in SKBR3 cells.

These results were also independently confirmed in live cell experiments using the same quantification method for PtdIns(3,4,5)P_3_. That is, PtdIns(3,4,5)P_3_ levels increased upon stimulation at the PM and were reduced below basal level upon PI3K inhibition in NIH3T3 but not in SKBR3 cells (Fig. S7).

PtdSer levels in both cell lines were also quantified using a genetically encoded fluorescently labelled PtdSer sensor^32^ (mCherry-Lact-C2) fused to a self-cleaving P2A peptide and to GFP^33^. This way mCherry-Lact-C2 and free-diffusing GFP were expressed at the same rate, allowing ratiometric imaging of the sensor. We did not observe any growth factor stimulation-related differences in localisation to the PM of the PtdSer biosensor (Fig. S8). These data demonstrated that the levels of PtdSer were not significantly modified by stimulation or inhibition of the PI3K pathway in both cell lines.

### In dysregulated PI3K cells, PDK1 homodimerisation and localisation was independent of its PH binding to PtdIns(3,4,5)P_3_ and PtdIns(3,4,5)P_3_ levels

Having measured the changes in PtdIns(3,4,5)P_3_ levels between the two cell lines, we next monitored the dynamic regulations of PDK1 in SKBR3 cells, where a different behaviour from NIH3T3 cells was identified. All PDK1 variants showed a strong constitutive association with the PM (Fig. 3A,B); this was also consistent for the PtdIns(3,4,5)P_3_ binding-defective (K465A) and the R466A/K467A PDK1 mutants. PI3K inhibition prior to stimulation did not affect the recruitment of any of the PDK1 variants to the SKBR3 PM (Fig. 3B). A prominent PM localisation was observed for the homodimer conformers, including mutant R466A/K467A-PDK1, whereas in NIH3T3 cells, these were localised preferentially in the cytoplasm (Fig. 2B). Our results had indicated that in NIH3T3 cells the localisation of PDK1 (irrespective of its dimerisation status) was dependent on the transient production of PtdIns(3,4,5)P_3_ at the PM (Fig. 2B), while in SKBR3 cells PDK1 localisation was constitutively at the PM and did not change significantly, either through PDK1 PH domain mutation (K465A) or upon PI3K stimulation or inhibition (Fig. 3B). This suggested that the overall localisation of PDK1 was independent of the transient formation of PtdIns(3,4,5)P_3_. Furthermore, the double mutation R466/K467 to alanine did not prevent PDK1 from translocating to the PM in SKBR3 nor in NIH3T3 cells, indicating events other than anionic lipids binding to the PH domain were involved in PDK1 recruitment.

Our next step entailed interrogating how the constitutive PM localisation would influence the formation of PDK1 homodimers in dysregulated SKBR3 cells. Figure 3C shows that WT-PDK1 responded to epidermal growth factor (EGF) stimulation shown by the increase of the homodimer population. However, PI3K inhibition did not prevent EGF-stimulated increase in WT-PDK1 homodimers (Fig. 3Cc). This result was not due to a lack of effect of the PI3K inhibitor, since it blocked EGF-induced PtdIns(3,4,5)P_3_ increase in SKBR3 cells (Fig. S6D,E and S7). It rather implied that, while the increase in homodimers was dependent on EGF stimulation, it was independent of transient formation of PtdIns(3,4,5)P_3_. The unexpected behaviour of the PDK1 mutants showed a significant increase in the basal homodimerisation compared to WT-PDK1 (Fig. 3C).

The fact that PDK1 was able to homodimerise independently of variations in PtdIns(3,4,5)P_3_, and that none of the PH domain mutations affected the binding of full-length PDK1 to the PM, pointed towards a critical role for the kinase domain in the regulation of PDK1 localisation and dynamics. To test this hypothesis, we investigated how isolated PDK1 PH domains in SKBR3 cells would recruit and dimerise at the PM. To this end, the cells were co-transfected with GFP-PH^PDK1^ and mRFP-PH^PDK1^ (Fig. 4A). The isolated WT PH^PDK1^ localised at the PM analogous to full-length PDK1 in these cells (Fig. 4B). However, contrary to the full-length protein, their relative PM/Cytoplasm distribution was modulated by PtdIns(3,4,5)P_3_ varying levels upon growth factor stimulation and PI3K inhibition. The K465A PH-domain mutant did not respond to increased PtdIns(3,4,5)P_3_ as expected from the *in-vitro* experiments. All the mutations affected the localisation of the PH domains to the PM, with the mutation R466A/K467A having the strongest effect (Fig. 4B).

Direct assessment of the wild type PH^PDK1^ homodimerisation in SKBR3 cells showed that, contrary to the full-length protein (Fig. 3C), PH^PDK1^ homodimerisation was strictly PtdIns(3,4,5)P3-dependent (Fig. 4C). EGF stimulation induced the production of WT-PH^PDK1^ dimers, inhibited by pretreatment with LY294002. The loss of PtdIns(3,4,5)P_3_ binding in the K465A mutant prevented the formation of homodimers upon stimulation. Similar to the wild type, the activating mutant PH domain (T513E) dimers were formed upon stimulation. The mutation of the R466/K467 residues totally abolished the basal dimerisation of the PDK1 PH domains. The PH domain homodimer population (Fig. 4D) was localised at the PM and was reduced by LY294002 treatment or mutation of the PH domain. The loss of the homodimers at PM was most significant in the case of the R466A/K467A-PH^PDK1^ mutant.

These data revealed that the dynamic regulation of the isolated PH domains of PDK1 was different from the regulation of full-length PDK1 in SKBR3 cells. PH^PDK1^ variants homodimerised to a level that was consistent with their localisation at the PM and the transient production of PtdIns(3,4,5)P_3_ (Fig. 4B–D), whereas the full-length protein did not behave in this manner (Fig. 3C). Therefore, these results indicated that the PH domain was not the sole domain with a role in the localisation of full-length PDK1 at the PM or its homodimerisation, highlighting the critical contribution of the kinase domain in the modulation of PDK1 conformational dynamics. Furthermore, the differences between the full-length PDK1 and isolated PH domain illustrated that, in SKBR3 cells, PDK1 was not exclusively regulated by the acute formation of PtdIns(3,4,5)P_3_. Instead, other anionic phospholipids would concur to the regulation of PDK1 in a kinase domain-dependent manner.

Our data demonstrates that PDK1 dimerisation by the modulation of PtdIns(3,4,5)P_3_ levels was the major determinant of PDK1 activity in NIH3T3 cells (Fig. 2C,E). However, in SKBR3 cells, where the PI3K pathway is dysregulated, the formation of PDK1 dimers did not concur with PtdIns(3,4,5)P_3_ variations (Fig. 3C). We suggest that the conventional model for the regulation of PDK1 activity by the transient production of PtdIns(3,4,5)P_3_ is incomplete, as it does not account for the regulation of PDK1 in cells with a dysregulated PI3 kinase pathway.

### PDK1-Akt/PKB heterodimerisation and downstream activation of Akt/PKB correlates with PDK1 homodimer formation in SKBR3 cells

To determine how the aberrant production of PDK1 homodimers in SKBR3 cells influenced downstream substrates activation, we monitored the phosphorylation of two PDK1 substrates Akt/PKB and SGK1. Previously, we had shown that Akt/PKB activation required PDK1-Akt/PKB heterodimerisation after binding to PtdIns(3,4,5)P_3_^10^. We proceeded in determining their interaction by coexpressing, in SKBR3 cells, eGFP-myc-PDK1 and mCherry-Akt/PKB (Figs 5A and S2C). We determined that the population of PDK1-Akt/PKB heterodimers (Fig. 5B) correlated with the formation of PDK1 homodimers observed in Fig. 3C. Similar to PDK1 homodimerisation, the heterodimer population of WT-PDK1-Akt/PKB increased upon EGF stimulation. The PtdIns(3,4,5)P_3_ binding-defective mutation K465A did not prevent the heterodimerisation of PDK1 with Akt/PKB. Following the same pattern than WT-PDK1, the interaction of T513E-PDK1 with Akt/PKB showed an enhanced heterodimerisation upon activation. We demonstrated the relevance of the R466A/K467A mutation in signalling propagation, since the binding of the R466A/K467A-PDK1 mutant to Akt/PKB was significant, again correlating with the elevated *E*_D_ of the PDK1 homodimer detected for the same mutant (Fig. 3C).

The enhanced interaction of all the PDK1 variants with Akt/PKB upon stimulation led in every case to an increase of Akt/PKB phosphorylation at its activation loop T308 (Fig. 5C). On the contrary, PI3K inhibition inhibited Akt/PKB T308 phosphorylation upon stimulation. The PDK1 mutation K465A did not prevent the EGF-triggered increase in T308 phosphorylation, which was consistent with the fact that this mutation did not obstruct either the localisation of full-length PDK1 K465A to the PM (Fig. 3B) or the increase in PDK1-Akt/PKB heterodimerisation (Fig. 5B). Finally, a significant increase in T308 phosphorylation was observed with both the activating mutant T513E and the R466A/K467A mutant compared to WT-PDK1, specifically under basal conditions (Fig. 5Ca,g,j). This correlated with the fact that T513E increased PDK1 activity and that the R466A/K467A mutation induced the formation of PDK1-Akt/PKB heterodimers in basal conditions (Fig. 5B).

To test whether the activation of PDK1 due to the mutation of R466A/K467A could also impact more endogenous substrates other than Akt/PKB, we investigated the phosphorylation status of endogenous SGK1 at its T-loop (T256) (Figs 5D and S9). Activation of SGK1 is known to be dependent on PI3K activation and the production of PtdIns(3,4,5)P_3_ and requires its hydrophobic motif phosphorylation by mTORC2^34,35^. However, unlike Akt/PKB, the kinase itself does not have a PH domain to directly interact with PtdIns(3,4,5)P_3_^35^. Figure 5D shows a similar effect for SGK1: We observed the enhanced phosphorylation of SGK1’s T-loop upon stimulation with EGF in a PI3K-dependent manner upon expression of WT PDK1. Furthermore, as observed with Akt/PKB, PDK1 R466A/K467A mutant also increased the phosphorylation of SGK1 under basal and stimulated conditions (Fig. 5D).

Altogether, the data obtained in both NIH3T3 and SKBR3 suggest that, in addition to the well-established requirement for PtdIns(3,4,5)P_3_, the binding of PDK1 to other anionic phospholipids is also critical in the regulation of PDK1 downstream substrates. More specifically, while PDK1 interaction with PtdIns(3,4,5)P_3_ would trigger downstream substrate activation, the binding to anionic phospholipids through K466/R467 would be inhibitory.

### Molecular conformations of PDK1 PH domains homodimers binding to PtdIns(3,4,5)P3 and/or PtdSer

**Figure 6:**
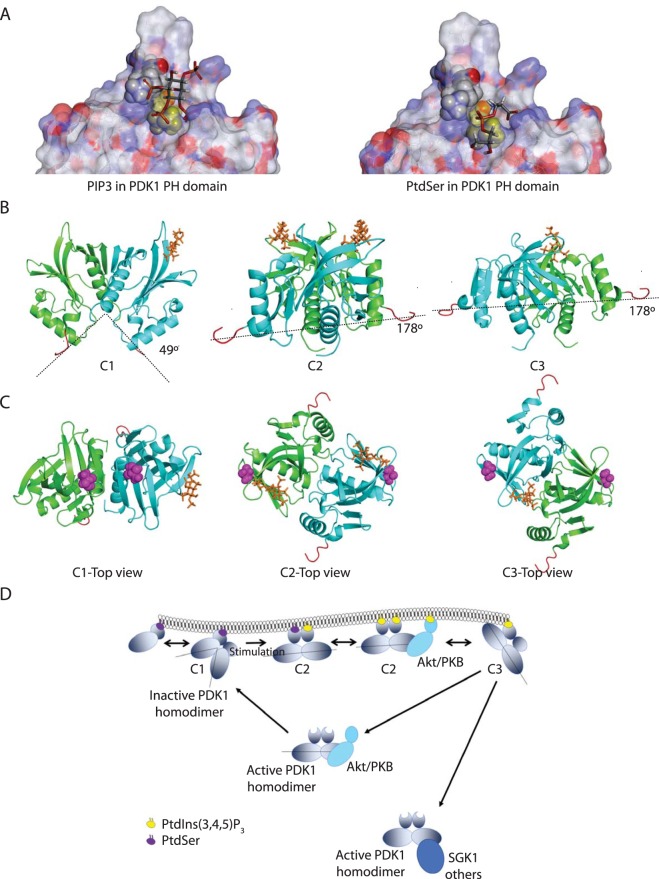
Molecular modelling of PDK1 PH domain homodimers and model of PDK1 homodimers activation. (**A)** Docking of PtdIns(3,4,5)P_3_ (left panel) and PtdSer (right panel) on PDK1 PH domain. Residue K465 is in yellow. **(B**,**C)** Representation of the three possible PDK1 PH domain homodimers bound to anionic phospholipids. **(D)** Model of the predicted active and inactive PDK1 homodimer conformers and their interaction with downstream substrates.

Molecular modelling and docking experiments were performed (Supplementary Information) to understand the molecular mechanisms whereby PDK1 dynamics would be differentially regulated by binding to PtdIns(3,4,5)P_3_ and PtdSer. This approach also determined the binding site for these anionic phospholipids. The binding of PtdIns(3,4,5)P_3_ and PtdSer were modelled on isolated PDK1 PH domain. The experiments showed that both anionic phospholipids recognised the same site on the PDK1 PH domain, albeit through different interactions and affinities (Fig. 6A). These results indicated that contrary to previous propositions^6^ the two phospholipids could not bind concomitantly to the same PH domain. The obstruction of PtdSer in the binding of PDK1 PH domains in GUV containing PtdIns(3,4,5)P_3_ (Fig. 1), was therefore unlikely to be due to the two phospholipids binding to the same PH domain. An alternative explanation was that PH^PDK1^ could bind to PtdSer and PtdIns(3,4,5)P_3_ as a dimer with each PH monomer binding to a different phospholipid. By performing protein-protein docking experiments we identified three possible PH domain homodimer conformers (C1, C2 and C3) formed by interacting with different combination of PtdSer and PtdIns(3,4,5)P_3_ binding to the PH domain pocket (Fig. 6B,C). The unstructured N-terminal linkers that mediate association to the kinase domains are also presented in Fig. 6B to evaluate the positioning of the kinase domain in the various conformers (red segments). The C1 conformation is associated only with one PtdSer molecule bound to one of the PH domains in the dimer while the other PH domain remained unoccupied. In C1, the relative positioning of the two PH domains did not allow for the simultaneous binding of two phospholipids at the same time at the membrane. Furthermore, the localisation of the linkers to the kinase domains suggested a close proximity of the two kinases (see the closed angle of 49°). Similar to C1, the C3 conformer arises when only one lipid was bound to one PH domain in the dimer, but in the case of C3 the lipid was PtdIns(3,4,5)P_3_. Both C1 and C3 could potentially adopt a C2 conformation by binding to either another PtdSer or a PtdIns(3,4,5)P_3_ with the empty remaining PH domain. The C2 conformation was compatible with a full access to both lipid sites at the membrane. Contrary to the C1 conformation, the linkers in C2 were at maximum distance and angle (178°). This shows that the kinase domains in C2 were much further apart than in the C1 conformation, and could be in a position allowing the access of the substrates to the kinase active sites. In this condition, the C2 conformer would be compatible with an active conformation of the PDK1 homodimer. It is worth noting that while the angles between the conformers C2 and C3 are the same, the complexes bear a different geometry and might not have the same activity.

In light of these results, we suggest (Fig. 6D) that prior to stimulation, PDK1 homodimers would bind to the abundantly available PtdSer (and from our biochemical data possibly also PtdIns(4,5)P_2_) in an inactive C1 conformation. This event would permit PDK1 recruitment to the PM, but due to close proximity of the two kinase domains, C1 conformation would remain inactive. Upon growth factor stimulation and increase in PtdIns(3,4,5)P_3_ levels at the PM, the further interaction of C1 with PtdIns(3,4,5)P_3_ via its unoccupied PH domain would trigger a change in conformation towards an active C2 conformer. The separation of the two kinases in the C2 homodimer would allow accessibility to substrates and hence downstream activation. The molecular modelling data also predicted that both the binding of two PtdIns(3,4,5)P_3_ or a non-lipid-binding state (where both PH domains are empty) would also trigger the active C2 conformation of the PDK1 homodimers. These results were of great significance since they were compatible with our biochemical findings where both PtdIns(3,4,5)P_3_-triggered active PM homodimers as well as cytoplasmic (unbound) PDK1 homodimers, were capable of phosphorylating downstream substrates like Akt/PKB and SGK1.

The molecular mechanisms that would lead to the disassociation from the PM have not yet been identified. Our work and others suggest that it would likely involve the autophosphorylation of T513 and S410. We suggest that electrostatic repulsion would be a likely mechanism for triggering the loss of electrostatic binding to anionic phospholipids. Our molecular modelling data (Table 2) showed that the phosphorylation of T513 prevented the formation of the C1 conformer, suggesting that the presence of phospho-T513 would not be compatible with an inactive conformation of the PDK1 homodimer. Table 2 also shows that in the C3 conformer both S410 and T513 are closer to the inositol binding site than other conformations. This suggests that C3 might be a transition conformer that would occur following the loss of binding to one of the PtdIns(3,4,5)P_3_. The autophosphorylation of the residues S410 and T513 in the C3 (positioned closer to the anionic phospholipid binding site than in other conformations) could trigger repulsion from the PM.

**Table 2:** Distances between residues approximated to the closest number.

Monomer A-Monomer B distances (Å)	C1	C2	C3
Ser410 – Binding pocket	44	33	31
Ser410 – Thr513	40	39	41
Thr513 – Binding Pocket	30	37	29
Thr513 – Thr513	15	48	43
Ser410 – Ser410	31	52	64

## Discussion

Previously, we had identified PDK1 homodimerisation as a central component of its acute regulation and, showed, by *in situ* FRET-FLIM, that PDK1 homodimerised in the cytoplasm of COS7 cells in a PtdIns(3,4,5)P_3_ dependent manner^13^. In that work we suggested that the autophosphorylation of T513 upon stimulation might lead to the production of active PDK1 monomers through disruption of the inactive homodimers^13^.

The current refinement of our FRET-FLIM methodology^23^ permitted a deeper understanding of the complex mechanisms underpinning the regulation of PDK1. By monitoring the conformational dynamics of the PDK1 T513E mutant *in situ* in NIH3T3 (Fig. 2C) and SKBR3 cells (Fig. 3C) we observed that the T513E mutation did not lead to the disassembly of the PDK1 homodimers, but generated an increase in a homodimer population correlating with the activation of its downstream substrates (Akt/PKB and SGK1) (Figs 2C,E and 5C,D). This may be interpreted in two ways. Firstly, that the PDK1 conformer requires a transition to an activated homodimer conformation prior to downstream substrate phosphorylation. Secondly, that PDK1 homodimerises in more than one conformation. We suggest that PDK1’s autophosphorylation at the T513 residue, triggered upon PM translocation, could be a switch or a stabilising factor of the conformational change. Work by Kang *et al*. suggested, using co-immunoprecipitation of the isolated PDK1 PH and kinase domains, the presence of more than one possible PDK1 homodimer conformation^22^. They proposed a key role for the residue T513 in the formation of different dimers.

Our recent quantification of the PDK1 homodimerisation affinity, determined by the effective intracellular dissociation constant ${K}_{D}^{eff}$ of PDK1 homodimerisation in SKBR3 cells, also demonstrated the presence of more than one homodimer conformation^23^. In this latter work, PDK1 homodimerisation was detected by time resolved-FRET between GFP- and Cherry-tagged PDK1. From these measurements, we established that the effective intracellular dissociation constant at basal conditions ((${K}_{D}^{eff}$)_basal_ = 49 µM) was greater than post stimulation ((${K}_{D}^{eff}$)_EGF_ = 11 µM). The explanation for the decrease in the effective *K*_D_ upon stimulation could be the contribution of a FRET-inactive PDK1 homodimer population (a dimer population that does not FRET), versus the FRET-active PDK1 homodimer population. The FRET-inactive dimer would correspond to a conformer geometry unfavourable to the efficient transfer of energy. This can issue from the variability in the orientation of the chromophores, a parameter which, in addition to the distance between FRET partners, also defines efficient transfer of energy. The results from the de Las Heras paper^23^ support the idea of PDK1 homodimer species with different conformations, coexisting at equilibrium with their relative abundance being dependant on the activation signal.

We suggest that the homodimer conformers with different topologies are a more effective mechanism of regulating PDK1 rather than the pre-assumed monomeric forms. This effective mechanism may provide a refined control of PDK1 activity by permitting a broader array of anionic phospholipids binding to the various topologies. The occurrence of inactive PDK1 homodimers could be explained by a transition ‘primed’ conformer species: PDK1 has been shown to be constitutively autophosphorylated on its activation loop (S241) independently of growth factor stimulation^18^. Therefore, in its inactive conformation, the PDK1 homodimer would be phosphorylated on the activation loop but auto-inhibited prior to stimulation. This would allow for a rapid activation of PDK1 upon growth factor stimulation. Such behaviour is well established in other AGC kinases, such as PKCs. The members of this family are fully phosphorylated (primed) at their activation sites but autoinhibited by a pseudosubstrate sequence prior to stimulation^36^. Our molecular model (Fig. 6) provides the possible conformation of an inactive PDK1 homodimer, prior to PtdIns(3,4,5)P_3_ binding and autoinhibitory configuration where the close proximity of the kinases would hinder the interaction with substrates (Fig. 6 dimer C1). The possibility of dimeric PH domains allows a combinatory binding of various anionic lipids that would not be feasible with monomeric PDK1, thus providing a refined regulatory step for its activation.

By exploiting the spatial and conformational dynamics of PDK1 and its PH domain mutants (K465A and R466A/K467A), we identified the differential role of the binding of anionic phospholipids as a molecular switch of the inactive to the active PDK1 conformations. The *in vitro* affinity measurements of isolated PDK1 PH domains indicated that the affinity for PtdSer is two to three orders of magnitude lower than for PtdIns(3,4,5)P_3_ (Fig. 1). We showed that the mutation K465A inhibits the binding to PtdIns(3,4,5)P_3_ and PtdIns(4,5)P_2_ but not PtdSer, and that, conversely, the mutation R466 A/K467A prevents the interaction of PtdSer and PtdIns(4,5)P_2_, but not PtdIns(3,4,5)P_3_. Therefore, despite the close proximity of the residues K465, R466 and K467 in PDK1 PH domain, the role of different anionic phospholipids in cells can be distinguished by using these differential mutations. The results from the sFCS experiments confirm the specific binding of PDK1 PH domain to PtdIns(3,4,5)P_3_ containing GUVs. The presence of PtdSer in the same GUV interfered with the PH^PDK1^ binding to PtdIns(3,4,5)P_3_, suggesting that the close proximity of PtdSer to the PtdIns(3,4,5)P_3_–bound PH^PDK1^ facilitated the association of PtdSer. Since our molecular model (Fig. 6A) established that the docking cavity cannot simultaneously accommodate PtdIns(3,4,5)P_3_ and PtdSer as previously proposed^6^, our results suggest that PDK1 may associate to both lipids as a dimer, with each PH unit binding a different lipid.

The weaker *in-vitro* affinity of the PDK1 PH domain for PtdSer compared to PtdIns(3,4,5)P_3_, raises the question whether, in the cell, the PtdSer-PH domain interaction is still strong enough to recruit PDK1 to the plasma membrane. In unstimulated cells (low PIP3 levels), K465A PH^PDK1^, binds PtdSer but not PtdIns(3,4,5)P_3_ and was distributed at the plasma membrane, albeit at a slightly lower level than WT PH^PDK1^ (Fig. 4B). As expected, K465A PH^PDK1^ did not associate to the membrane upon PtdIns(3,4,5)P_3_ increase. On the other hand, the PtdSer binding-defective mutant, R465A/K466A PH^PDK1^, did bind PtdIns(3,4,5)P_3_ *in-vitro* (Fig. 1A) and in the cell, and homodimerized upon the increased levels of PtdIns(3,4,5)P_3_ (Fig. 4C). This mutant (R465A/K466A) was recruited to the plasma membrane (Fig. 4B) but at much lower levels than all other PH^PDK1^ variants. This highlights the relevance of the PtdSer - PH domain interaction, implying that it has a significant contribution in recruiting the PDK1 to the PM even in low PtdIns(3,4,5)P_3_ conditions. We note, however, than besides this interaction, when considering the full PDK1 protein, it is also important to take into account the role of the kinase domain in PDK1 intracellular localisation, as shown in Figs 3B and 4B.

Our model demonstrates that PtdIns(3,4,5)P_3_ and PtdSer bind to a PDK1 PH-PH dimer, with each anionic phospholipid binding to one PH domain. Moreover, it predicts the formation of three types of PH^PDK1^ homodimers with various propensities to interact with anionic phospholipids. Briefly, the C1 conformer binds to PtdSer (only one of the two PH binds); C2 conformer binds to two anionic phospholipids, (PtdSer-PtdIns(3,4,5)P_3_) or (PtdIns(3,4,5)P_3_-PtdIns(3,4,5)P_3_); the C3 conformer binds only to PtdIns(3,4,5)P_3_ (only one of the two PH binds).

In addition to the regulation by anionic phospholipids, the post-translational modification of the PH domains by T513 phosphorylation can affect PDK1 activation in a PH domain dependent manner. Binding affinity assays and sFCS data showed that the activating PH domain mutation T513E induced the loss of PH^PDK1^ specific binding to PtdIns(4,5)P_2_ and PtdSer and a strong reduction to PtdIns(3,4,5)P_3_ (Fig. 1B,D), therefore suggesting that the activating effect of T513 phosphorylation would be through the disassociation of PDK1 from the PM, thereafter promoting PDK1 interaction with its downstream substrates.

By monitoring the localisation and the conformational dynamics of full-length PDK1 in NIH3T3 cells, by FRET-FLIM, we confirmed the interplay of the anionic phospholipids. PDK1 translocation to the PM upon stimulation induced a PtdIns(3,4,5)P_3_ binding-dependent increase in homodimerisation. This increment was correlated with an increase in downstream substrate phosphorylation. These results suggested that activated PDK1 homodimers upon stimulation were involved in the activation of the downstream substrates. We observed that the loss of PM binding (R446A/K467A mutant), also elicits a strong constitutive dimerisation of PDK1 promoting downstream substrates phosphorylation (Fig. 2C,E). These data imply that PDK1 homodimer formation occurs through two independent mechanisms; one dependent on the binding to PtdIns(3,4,5)P_3_ at the PM and the other in response to the loss of the PM binding (through the R446A/K467A mutation). In both cases, the formation of PDK1 homodimers is correlated with the activation of its downstream substrate Akt/PKB. This suggested that these homodimers are similar in conformation and their activation state. We propose that the binding to PtdIns(3,4,5)P_3_ is responsible for the activation of PDK1 by forming PDK1 activated homodimers. Conversely, the anionic phospholipids binding to residues R466/K467 prior to PtdIns(3,4,5)P_3_ formation (in basal conditions), are therefore responsible for maintaining PDK1 in an inactive conformation.

The abundance of PtdSer and PtdIns(4,5)P_2_ and the fact that PtdSer can cooperate with PtdIns(3,4,5)P_3_ to target PDK1 PH domain to the PM (Fig. 1D), as observed in other PH domains^37,38^, suggest that these anionic phospholipids are responsible for the negative regulation of PDK1 through binding to R466/K467. Furthermore, the R466A/K467A mutant resulted in a loss of its specific cellular localisation without a significant effect on the formation of total dimer population. This implies that the homodimers of PDK1 are differentially regulated by anionic phospholipids, a prerequisite for the refined regulation of PDK1. The association with PtdSer (and possibly PtdIns(4,5)P_2_) increases the membrane residence time of the full-length protein and may affect its affinity for PtdIns(3,4,5)P_3_. These effects could provide PDK1 with a search mechanism for PtdIns(3,4,5)P_3_, similar to that proposed for GRP1^PH^ ^39^. This regulation mechanism would also be compatible with the micro-domain compartmentalisation during the initial stages of PI3K signalling and the constrained lateral diffusion exhibited by PDK1’s immediate downstream target Akt/PKB^40^. Besides, PtdSer may contribute to the clustering of the phosphoinositides, which could enhance the binding of PDK1 to the membrane, promoting dimerization of this protein.

To determine the effect of the dysregulation of the PI3K pathway on the regulation of PDK1, we utilised SKBR3 cells that have constitutively elevated PtdIns(3,4,5)P_3_ levels. We showed that PDK1 regulation was perturbed in this cell line. Growth factor stimulation still resulted in activated PDK1 homodimer formation (Fig. 3C). Binding and activation of the downstream effectors Akt/PKB and SGK1 (Fig. 5C,D) was also triggered. However, the inhibition of the PI3K pathway due to the pre-elevated PtdIns(3,4,5)P_3_ levels did not affect dimer formation, suggesting that under dysregulated conditions, PtdIns(3,4,5)P_3_ does not behave as the molecular switch. Essentially, all mutants K465A, R466A/K467A and T513A elicited a PDK1 homodimerisation (Fig. 3C), which correlated with downstream substrates activation (Fig. 5C–D), indicating the formation of activated homodimers. The overall population of PDK1 homodimers was mainly located at the PM, irrespective of the PH domain mutations (Fig. 3D). This suggested that the kinase domain had a significant influence on the localisation and dynamics of a cellular environment exhibiting a dysregulated PI3K pathway (SKBR3 cells). Contrary to the full-length PDK1, the PH^PDK1^ wild type and its mutants behaved as predicted in a regulated PI3K pathway (as in NIH3T3 cells) (Fig. 4). Therefore, the kinase domain is a major determinant in the regulation of PDK1 not only in terms of conformational dynamics but also through its localisation at the PM.

This finding corroborates with Komander *et al*. who have also suggested that full-length PDK1 localisation in cells did not strictly follow the occurrence of PtdIns(3,4,5)P_3_ at the PM unlike the isolated PDK1 PH domains^5^. They postulated that PDK1 could be sequestered in the cytosol possibly by binding to soluble inositol phospholipids IP5/IP6. It is not clear whether this sequestration would involve the binding of monomers or dimers of PDK1, and how the presence of the kinase domain would influence this sequestration, but it could be part of the mechanism that distinguishes the behaviour of PDK1 in SKBR3 from NIH3T3 cells.

In summary, our results reveal that PDK1 homodimerises in response to an increase in PtdIns(3,4,5)P_3_ levels and that the activated PDK1 dimers phosphorylate both membranous (Akt/PKB) and cytoplasmic (SGK1) substrates. Our observations establish the cooperative association of PtdSer with PtdIns(3,4,5)P_3_ in recruiting PDK1 to the PM, but they have opposite effects on PDK1 activation. Our results indicate that membrane-binding to anionic lipids other than PtdIns(3,4,5)P_3,_ would stabilise PM-associated PDK1 in a homodimer conformation where the catalytic site would be sequestered from potential substrates. The enhancement in PtdIns(3,4,5)P_3_ levels would induce a dual binding of the PDK1 homodimer from PtdSer only (C1, Fig. 6) to PtdSer- PtdIns(3,4,5)P_3_ or PtdIns(3,4,5)P_3_-PtdIns(3,4,5)P_3_ and a change in conformation of PDK1 consistent with an activated conformation (C2, Fig. 6). The phosphorylation of T513 leads to a transition C3 conformation and dissociation from the membrane where the PDK1 dimer adopts the C2 type activated conformation.

Finally, 41 missense substitutions had been found in PDK1 linked to various types of cancers (Cosmic database). Correlated to our results, one of these residues (R466) was found mutated 3 times; in brain (R466Q), breast (R466Q) and colon (R466W)^41^. Our findings indicate that a physiological alteration of the R466/K467 site in cells mimicking the effect of the mutant 466 A/467 A, and altering the binding to PtdSer or PtdIns(4,5)P_2_, would lead to the constitutive activation of PDK1 and potentially to a significant effect in different types of cancer. Therefore, detailed characterisation of this site, and its previously unappreciated role in PDK1 downregulation, would enable defining a novel therapeutic strategy where a constitutively activated dimer of PDK1 could be rendered inactive by small molecules that would drive its conformation towards an inactive membrane-bound conformer.

## Materials and Methods

### Reagents and antibodies

LY294002 in solution, recombinant human epidermal growth factor (EGF) and Mowiol 4–88 were purchased from Calbiochem. Recombinant human platelet-derived growth factor (PDGF), Lipofectamine LTX & Plus Reagent, penicillin/streptomycin (P/S), Sodium Pyruvate, GlutaMax Supplement and cell culture media were obtained from Life Technologies. Bovine serum albumin (BSA), sodium borohydride and the rest of reagents were purchased from Sigma-Aldrich. Antibodies against total AKT (mouse panAKT #40D4), phospho-AKT (rabbit pThr308 #244F9 and rabbit pSer473 #D9E), phospho-PKC(pan) (rabbit γThr514 #9379) and HA- and myc-tags (mouse #6E2 and rabbit #71D10, respectively) were purchased from Cell Signaling Technology; the antibody against tubulin (mouse α-Tubulin) was purchased from Sigma-Aldrich. Secondary antibodies for infrared detection (IRDye 680RD anti-mouse #926-32211, and IRDye 800CW anti-rabbit #926-68070) and the corresponding blocking buffer were obtained from LI-COR Inc (Nebraska). Glass-bottomed #1.5 35-mm dishes were obtained from MatTek Corporation (MA).

### Lipids

1,2-di-(9Z-octadecenoyl)-sn-glycero-3-phosphocholine (18:1 DOPC), 1-hexadecanoyl-2-(9Z-octadecenoyl)-sn-glycero-3-phospho-L-serine (18:1-16:0 POPS) and 1,2-di-(9Z-octadecenoyl)-*sn*-glycero-3-[phosphoinositol-3,4,5-trisphosphate (18:1 PtdIns(3,4,5)P_3_) were purchased from Avanti Polar Lipids (Alabaster, AL).

### Constructs

The eGFP-myc-PDK1, HA-PDK1-mCherry were cloned as described elsewhere^13^.

mRFP-HA-PKB constructs were cloned as described elsewhere^13^.

GFP-Lact-C2 was obtained from AddGene. Cloning of the K465A and R466A/K467A mutants of PDK1 and PH^PDK1^, mRFP-HA-PKB, mCherry-6xGly-GFP, mCherry-LactC2-P2A-GFP, and GRP1^PH^ and Lyn11-FKBP2-mRFP is described in detail in the supplementary methods.

### Cell culture

Transfection, serum starvation procedure and sample preparation methods are described in the supplementary methods.

### Vesicle preparation

Giant Unilamelar Vesicles were prepared by spontaneous swelling as described in^25^.

### Western blots

Quantification of IR Western blots was done on an Odyssey Imaging system (Li-Cor Inc, Nebraska). Antibodies against panAKT and α-Tubulin were used as internal control for the quantification of Akt phosphorylation and SGK1 phosphorylation, respectively. Lysis conditions for western-blotting and procedures are described in the supplementary methods.

### Confocal imaging and FRET-FLIM

Confocal and lifetime images were acquired on a Leica TCS SP5 confocal scanning microscope through a 63×/1.30 objective. Samples were excited using a 488 nm and 543 CW lasers and fluorescence images were acquired in the 500–550 and the 575–750 nm windows. Lifetime imaging was performed using a TCSPC system (SPC-830, Becker & Hickl, Germany) with a pulsed laser (Mai-Tai, Spectra-Physics) tuned at 890 nm for excitation. Time-resolved fluorescence detection was performed in the 500–550 nm. Further information on the set-up can be found in the Supplementary Information. Experimental and analytical procedures for image segmentation, *E*_D_ quantification of the accessible PtdIns (3,4,5)P_3_ and PtdSer and calculation of the PM to cytoplasm ratio are detailed in the Supplementary Methods.

The position of the GFP and mCherry in PDK1 was relevant for the FRET measurements. Tagging PDK1 at the at the same terminal resulted in low FRET efficiency, presumably due to an unfavourable dimer geometry since our previous work had pointed towards a homodimer conformation where the N and C termini of the constituent monomers would be in close proximity to each other^13^.

### Scanning fluorescence correlation spectroscopy (sFCS)

sFCS is a single-molecule sensitivity technique that allows the diffusion coefficient of a molecule to be determine as the membrane-bound molecule diffuses on the surface of a vesicle^42,43^. sFCS was applied to study the diffusional behaviour and intermolecular interactions of fluorescently-labelled proteins bound to GUVs (Fig. S1A). A water immersion objective HCX PL APO 63×/1.20 W CORR Lbd Bl (Leica) on a confocal Leica SP5 was employed and confocal detection of the emission using the avalanche photodiodes fitted to the DD X1 output port of the microscope. To excite eGFP tagged species we used the 488 nm line from an Ar^+^ laser. The average power density at the sample plane was 50 kW/cm^2^. eGFP emission was detected through a 500–530 nm bandpass filter. Photon arrival times were registered using an SPC-830 TCSPC card (Becker & Hickl, Germany), which also registered the pixel, line and frame signals from the scanner. An HRT-82 Eight Channel Router (Becker & Hickl, Germany) was required to spectrally tag the signal of the detected photons. An external clock signal at 20 MHz was used (Fig. S1B)

The imaging mode at the microscope was set to *xt* (line-scanning) and the scanning frequency was 1400 Hz. The scanning length spanned over 16.64 μm, binned in 64 pixels of 260 nm each. The equatorial section of the GUVs was scanned for 5 min. The Becker & Hickl files encoding the arrival of the photons were decoded using an in-house Matlab routine (available at request). The *xt* photon trace was aligned to correct for the vesicle drift (Fig. S1C) and the resulting photon time trace was fit to a 2D diffusion model with a minimal lag-time given by the inverse of the scanning frequency:

1 $$G(\tau )=\frac{1}{N}{(1+\frac{\tau }{{\tau }_{D}})}^{-1/2}{(1+\frac{\tau }{{S}^{2}{\tau }_{D}})}^{-1/2}$$

where N is the average number of molecules in the focal volume, *τ* is the lag-time,* τ*_*D*_ is the diffusion time of the molecule and *S* ratio of the axial (*z*_o_) to the radial (beam waist, *ω*_o_) 1/e^2^ dimensions of the confocal volume. *ω*_o_ and *z*_o_ were obtained after calibrating the volume using a dye with known diffusion (Alexa 488, *D* ≈ 430 μm^2^/s at 25 °C). Finally, the diffusion coefficient is *D* = *ω*_o_^2^/(4τ_D_). All uncertainties are indicated as SEM.

### Molecular Modelling

Molecular modelling is described in the supplementary methods.

## Supplementary information

A Complex Interplay of Anionic Phospholipid Binding Regulates 3’-Phosphoinositide-Dependent-Kinase-1 Homodimer Activation
