# Supplementary material for: A Complex Interplay of Anionic Phospholipid Binding Regulates 3′-Phosphoinositide-Dependent-Kinase-1 Homodimer Activation
Source: Sci Rep. 2019 Oct 10;9:14527. doi: 10.1038/s41598-019-50742-8 (PMC6787260; doi:10.1038/s41598-019-50742-8)
Supplement: Supplementary file 1 — A Complex Interplay of Anionic Phospholipid Binding Regulates 3’-Phosphoinositide-Dependent-Kinase-1 Homodimer Activation [file 41598_2019_50742_MOESM1_ESM.pdf]

A Complex Interplay of Anionic Phospholipid Binding Regulates 3'-Phosphoinositide-Dependent-Kinase-1 Homodimer Activation

Supplementary Information

Gloria de las Heras-Martínez[1,4*], Véronique Calleja[2*], Remy Bailly[3], Jean Dessolin[3], Banafshé Larijani[4,5 $], Jose Requejo-Isidro[1,6, 7 $]

[1] Instituto Biofisika (CSIC, UPV/EHU), 48490 Leioa, Spain

[2] Protein Phosphorylation Laboratory, The Francis Crick Institute, 1 Midland Road, NW1 1AT, London, UK

[3] Institute of Chemistry & Biology of Membranes & Nanoobjects (UMR 5248 CBMN) CNRS – Université de Bordeaux - Bordeaux INP All. Geoffroy Saint-Hilaire, 33600 Pessac, France

[4] Cell Biophysics Laboratory, Ikerbasque Basque Foundation for Science, Instituto Biofisika (CSIC, UPV/EHU) & Research Centre for Experimental Marine Biology and Biotechnology (PiE), University of the Basque Country (UPV/EHU), Leioa 48940, Spain.

[5] Centre for Therapeutic Innovation (CTI-Bath); Cell Biophysics Laboratory Department of

Pharmacy & Pharmacology University, Bath, Claverton Down, Bath, BA2 7AY, United Kingdom

[6] Centro Nacional de Biotecnología (CSIC). Darwin, 3, E28049 Madrid, Spain

[7] Unidad de Nanobiotecnología, CNB-CSIC-IMDEA Nanociencia Associated Unit, 28049 Madrid, Spain

* Authors with equal contribution

$ To whom correspondence should be addressed: Prof. Banafshé Larijani: banafshe.larijani@ikerbasque.org & Dr Jose Requejo-Isidro: jose.requejo@csic.es

Supplementary Methods

- Cloning
- Cell culture and transfection
- Western blots and quantitative IR western blots
- Protein purification
- Protein lipid overlay assay
- Confocal and time-resolved imaging
- Preparation of fixed samples for time-resolved FRET experiments
- Image segmentation and calculation of the plasma membrane to cytoplasm partition coefficient
- Quantification of accessible PtdIns(3,4,5)P_3 and PtdSer
- Quantification of the Dimerisation Efficiency (E_D)
- Segmentation of FRET-FLIM images
- Molecular Modelling

Supplementary Figures

- Fig. S1: Experimental implementation of scanning FCS.
- Fig. S2: Fluorescently-tagged PDK1, PH^{PDK1} and Akt/PKB constructs.
- Fig. S3. Fractions of GFP-PHPDK1 stained with Coomassie following SDS-PAGE.
- Fig. S4: Quantification of the binding efficiency of two proteins using Time-Resolved FRET (FRET-FLIM).
- Fig. S5: Calculation of the plasma membrane to cytoplasm partition coefficient.
- Fig. S6: Quantification of PtdIns(3,4,5)P_3 levels in intact NIH3T3 and SKBR3 cells using the high-selectivity PH domain probe eGFP-$GRP1^{PH}$.
- Fig. S7: Time course of PtdIns(3,4,5)P_3 levels at the plasma membrane in live NIH3T3 and SKBR3 cells using eGFP-$GRP1^{PH}$.
- Fig. S8: Quantification of PtdSer levels at the plasma membrane of NIH3T3 and SKBR3 cells.
- Fig. S9: Use of phospho-PKC(pan) (γThr 514) to detect phosphorylation of endogenous SGK1 at its T-loop.

Supplementary Methods

Cloning. Both mutants K465A and R466A/K467A of human PDK1 were obtained by site-directed mutagenesis of the eGFP-myc-PDK1 and HA-PDK1-mCherry constructs[1] with the QuickChange mutagenesis kit (Agilent Technologies, Inc.). The oligos used for the mutagenesis were as follows: sense, 5'-ggcccagtggatgcgcggaagggtttatttgc-3' and antisense, 5'-gcaaataaacccttccgcgcatccactgggcc-3' for the K465A mutant and sense, 5'-ggcccagtggataaggcggcgggtttatttgcaag-3' and antisense, 5'-cttgcaaataaacccgccgccttatccactgggcc-3' for the R466A/K467A mutant. To obtain the constructs of the isolated PH domain of PDK1 (residues 404-556 at the C-terminus of PDK1), this domain was first amplified from the full-length eGFP-myc-PDK1 construct by PCR. The oligos were designed to introduce a myc tag sequence between the fluorescent protein and the PH domain (sense, 5'-ggaagatctgcaatggaacagaaactcatctctgaagaggatctgccccagaggtcaggc-3' and antisense, 5'-ctagtctagatcactgcacagcggcgtccgggtggctctggtatcg-3'). The whole myc-PDK1 sequence was removed from the original plasmid by enzymatic digestion with BglII and XbaI. The amplified myc-PH segment containing sites for BglII and XbaI at 5' and 3', respectively, was inserted in the digested plasmid to finally obtain: pCMV-eGFP-myc-PH^{PDK1}. The mRFP-myc-PH^{PDK1} was obtained by substitution of the eGFP by mRFP. To do so, we PCR amplified the mRFP sequence from the pCMV-mRFP-HA-PKB plasmid using two oligos that included EcoRI and BglII sites to remove the eGFP from the original plasmid by digestion with those enzymes: sense, 5'-ccggaattcatggcctcctccgaggacgtc-3' and antisense, 5'-ggaagatctagctgcaccggtggagtg. The K465A and R466A/K467A mutants of the isolated PH domain constructs were generated by site-directed mutagenesis using the same oligos as for the full-length protein. The mCherry-HA-PKB construct was cloned by PCR amplification of the mCherry sequence from the pCMV-HA-PDK1-mCherry plasmid with the oligos: sense, 5'-ccggaattcatggtgagcaagggcg-3' and antisense, 5'-ggaagatctcttgtacagctcgtccatgccgccg-3'. By enzymatic digestion with EcoRI and BglII of the original construct pCMV-mRFP-HA-PKB we removed the mRFP to insert the PCR product containing EcoRI-mCherry-BglII and finally obtain pCMV-mCherry-HA-PKB.

The constructs for the PH domain of GRP1 and its double mutant (K273A/R284A) were obtained by isolating the C-terminal part (residues 240-399) of the protein containing the PH domain (residues 263-380) from the original pTriEX6-versatile plasmids kindly provided by G. Chung (Cell Biophysics Lab, CRUK, London). The amplified C-terminus was inserted in a pCMV5 plasmid and fused to eGFP. The oligos for the PCR were as follows: sense, 5'-ggaagatctgaaagtatcaagaatgagc-3' and antisense, 5'-ctagtctagattatttcttattggcaatcctccttttc-3'.

mCherry-6Gly-eGFP were obtained from mCherry and eGFP containing plasmids. Three PCR were required to obtain mCherry-6Gly-eGFP: PCR1: sense, 5´-ctagctagcatggtgagcaagggcgaggaggataac-3´ and antisense, 5´-gctcaccatgccgccgccgccgccgcccttgtacagctcgtc-3´; PCR2: sense, 5´-tgtacaagggcggcggcggcggcggcatggtgagcaaggg-3´ and antisense, 5´-aaatatgcggccgctttacttgtacagctcgtccatgcc-3´. The products obtained from the first and second PCR were used as templates for a last step using the sense PCR1 and the antisense PCR2 primers. The resulting construct contains NheI and NotI sites at 5´and 3´ respectively. The Cherry-6Gly-eGFP fragment was now removed from the parental vector by enzymatic digestion using those sites and it was finally inserted into a pCMV backbone.

mCherry-LactC2-P2A-eGFP was obtained following a similar procedure to mCherry-6Gly-eGFP using mCherry-LactC2 [2] and mCherry-P2A-eGFP constructs as the templates for the initial reactions PCR1 and PCR2, respectively. The oligos were: PCR1: sense, 5´-ctagctagcatggtgagcaagggcgaggaggataac-3´ and antisense, 5´-cagcaggctgaagttagtagcacagcccagcagctcc-3´; PCR2: sense, 5´-ggagctgctgggctgtgctactaacttcagcctgctg-3´ and antisense, 5´-cccaagcttttacttgtacagctcgtccatgccgagagtgatc-3´. For the third PCR reaction, the sense PCR1 and antisense PCR2 oligos were used and the final template was digested with NheI and HindIII and inserted in a pCMV backbone.

Cell culture and transfection. SKBR3 and NIH3T3 cells were obtained from ATCC. SKBR3 and NIH3T3 cells were maintained in DMEM (Dulbecco's modified Eagle´s medium) with GlutaMAX containing 1% penicillin/streptomycin (P/S) and 10% foetal bovine serum (FBS) or donor calf serum (DCS), respectively, at 37°C and 5% CO_2 for SKBR3 or 10% for NIH3T3. For experiments, cells were seeded at 300,000 cells per well of a six-well plate (for Western Blotting) or per MatTek dish (for FRET/FLIM experiments).

Cells were co-transfected with 0.5 µg of donor DNA and 1 µg of acceptor DNA using Lipofectamine LTX & Plus Reagent in OptiMem containing GlutaMAX, following the protocol specified by the manufacturer. SKBR3 cells were incubated in this mixture for 4 h at 37°C and 5% CO_2 while NIH3T3 were incubated for 3 h at 37°C and 10% CO_2. The medium was removed and replaced by DMEM (with FBS or DCS and P/S) for another 3-4 h to let the cells recover. Finally, to serum starve SKBR3 cells the medium was replaced with DMEM containing only 0.2% of BSA and 1% P/S for at least 20 h. NIH3T3 cells were not serum starved. Experiments were performed about 24 h after transfection.

Western blots. Cells were seeded at 300,000 per well of a 6-well plate, transfected, serum-starved and stimulated as indicated above. After stimulation cells were washed twice in cold PBS and lysed for 5 min on ice in lysis buffer (20 mM Tris-HCl (pH 7.4), 150 mM NaCl, 100 mM NaF, 10 mM $Na_4P_2O_7$ and 10 mM EDTA supplemented with 1% (v/v) Triton X-100 and one complete protease inhibitor cocktail tablet (Roche)). After scraping, cells were centrifuged at 20,000g at 4°C for 10 min to remove cell debris and the lysis reaction was terminated by addition of 5x SDS loading buffer (250 mM Tris-HCl (pH 6.8), 10% (w/v) SDS, 40% (v/v) glycerol, 0.1% (w/v) bromophenol blue and 125 mM EDTA (pH 8.0)) supplemented with 10% β-mercaptoethanol. Samples were boiled for 5 min at 95°C. The proteins were separated on a NuPAGE 8.5% Bis-Tris Gel (Invitrogen) and transferred to a polyvinylidene difluoride (PVDF) membrane (Immobilon FL, Millipore) via a semi-wet process with the membrane first soaked in MeOH and then in transfer buffer (39 mM glycine, 48 mM Tris, 0.04% (w/v) SDS and 20% (v/v) MeOH). After the transfer, the membrane was incubated in blocking buffer (LI-COR) for 1 h and then for 4 h at R/T with the appropriate primary antibodies diluted in the blocking buffer at either 1:1000 dilution (pan Akt, pThr 308), 1:500 (γThr 514) or 1:1600 (α-Tubulin). After a first wash of the membrane (PBS with 1% (w/v) milk and 0.2% (v/v) Tween-20) to remove unspecific binding it was incubated for 1 h with the infrared dye-conjugated secondary antibodies (LI-COR) at a 1:5000 dilution each. The membrane was scanned on the Odyssey infrared imaging system (LI-COR) after a final wash.

Quantitative IR Western blots. Western blotting using fluorescent IR secondary antibodies allows two antibodies from different origin to be detected simultaneously. We used a pan antibody (pan Akt for the quantification of Akt phosphorylation and α-Tubulin for the quantification of SGK1 phosphorylation) and the phospho-antibody indicated in each Figure. The fluorescent signal registered for each phospho-antibody was normalised to its corresponding pan antibody after background subtraction.

Phosphorylation of SGK1 of its T-loop at Thr 256 was quantified using the phospho-PKC(pan) antibody, which has been previously shown to recognise phosphorylation at the T-loop of several AGC kinases including SGK isoforms, S6K and PDK1 (Fig. S9)[3,4]. The fluorescent signal was normalised to α-Tubulin signal.

Protein purification. The DNA sequences coding for the fluorescently tagged species of PH^{PDK1} were cloned in pRSET-6xHisTag-TEV vectors and purified by affinity chromatography. The pRSET plasmids were transformed in BL21 cells. The TEV site was included in the sequence to cleave the His tag after the initial purification using a TEV protease. A final polishing process was done by size-exclusion chromatography.

Protein lipid overlay assay. Commercial membrane lipid strips were purchased from Echelon (Salt Lake City, UT, USA). Otherwise, serial dilutions of PtdSer were spotted onto nitrocellulose membranes. In either case, the membranes were blocked for 1 h with a 3% BSA solution in TBS and they were incubated in 10 ml of a 30 µg/ml solution of recombinant eGFP-PH^{PDK1} species. Finally, the membranes were imaged using the fluorescent emission of eGFP to quantify the lipid-bound protein.

Imaging

Confocal and time-resolved imaging. Confocal and lifetime images were acquired on a Leica TCS SP5 confocal scanning microscope through a HCX PL APO 63x/1.30 GLYC CORR CS 21°C objective (276 nm pixel size).

Lifetime imaging was performed using 100 fs pulses from an 80 MHz rep-rate Ti:Sapphire laser (Mai-Tai, Spectra-Physics) tuned at 890 nm, with an average power at the sample of 1 mW. The emission in the 500-550 nm range was registered on a hybrid detector on non-descanned configuration using a TCSPC system (Becker & Hickl SPC-830; IRF 170 ps FWHM).

Confocal steady-state intensity images were acquired at 488 nm and 543 nm He:Ne, respectively, right after IR-excited lifetime imaging and after correcting the focal plane for chromatic aberration. The emission was detected at 500-550 nm (EGFP) and 575-750 nm (mCherry). The axial separation between the IR and the visible focal planes was calibrated upon IR and visible image registration at different planes, which yielded an average 1.2 µ separation between focal planes for the 63x/1.20W objective. The read-out for the fluorescence intensity was normalised to the excitation power and number of pixels in the region of interest of the image (Fluorescent Units). The average power density at the sample using CW visible lasers was 20 kW/cm^2.

Live cell imaging was performed on a confocal configuration using a HCX PL APO 63x/1.20 W CORR Lbd Bl objective at 68 nm pixel-size and setting the scanning speed at 8000 Hz (resonant scanning). During image acquisition cells were kept in a 5% CO_2 chamber at 37°C in observation medium (48 ml DMEM no phenol red (high glucose, no glutamine), 500 µl 100 mM Sodium Pyruvate, 500 µl of GlutaMax Supplement and 1 ml 10% BSA).

Preparation of fixed samples for time-resolved FRET experiments. Cells were seeded at 300,000 per MatTek dish and transfected as explained above. After the 20 h of serum starvation period SKBR3 cells were stimulated with EGF to a final concentration of 100 ng/ml for 2 min. Non-starved NIH3T3 were stimulated with PDGF (30 ng/ml) for 2 min. PI3K inhibition was done

prior to growth-factor stimulation treating the cells with 50 µM LY294002 for 20 min at 37°C and 5 or 10% CO_2, depending on the cell line. During growth factor stimulation cells were maintained at the same conditions, respectively. Immediately after, cells were twice washed in 2 ml cold phosphate-buffered saline (PBS) and fixed in 1 ml PBS containing 4% paraformaldehyde for 12 min at R/T. SKBR3 were washed again with PBS and incubated at R/T for 10 min in 2 ml of a solution of sodium borohydride in PBS at 0.1% (w/v) to minimize the autofluorescence from the cells. Dishes were washed again with 2 ml of cold PBS two or three times until the bubbles from the sodium borohydride solution disappeared. After completely removing the remaining PBS, the dishes were mounted with circular coverslips (#1.5, Menzel-Glässer) using 20 µl of Mowiol mounting medium (10% (w/v) Mowiol 4-88 in 25% (v/v) water, 25% (v/v) glycerol and 200 mM Tris-HCl (pH 8.5)) containing 2.5% (w/v) 1,4-diazabicyclo(2.2.2)octane (DABCO), an anti-fade reagent.

Image segmentation and calculation of the plasma membrane to cytoplasm partition coefficient. The confocal (visible excitation) and time-resolved (IR excitation) image of every cell was segmented into plasma membrane, cytoplasm and nucleus based on the confocal intensity images. The plasma membrane pixel-width was calibrated for every cell line and microscope resolution used on samples transfected with a membrane-bound probe (mRFP-Lyn or eGFP-PH^{PDK1}). This figure was later used to outline the membrane of cells with poor membrane to cytoplasm intensity contrast. All measurements were normalised to the number of pixels in each region to account for the effect of the different area of every region. Morphological effects were this way reduced and the membrane to cytoplasm partition coefficient was empirically found to be constant within roughly one micron of the equatorial section (Fig. S5). Cells were always imaged in this region to avoid relative intensity variability at the polar regions.

Quantification of accessible PtdIns(3,4,5)P_3 and PtdSer. To monitor and quantify intracellular PtdIns(3,4,5)P_3 we used the PH domain of GRP1, which binds PtdIns(3,4,5)P_3 with high specificity[5]. Cells were transfected with 0.4 µg of plasmid encoding a eGFP-$GRP1^{PH}$ construct and allowed to express for at least 24 h after transfection. SKBR3 cells were either fixed or kept alive in an incubating chamber (37°C/ 5% CO_2) after 20 h of serum-starvation. NIH3T3 cells were not serum-starved. Cells were imaged on the confocal microscope at 68 nm pixel size. Relative quantification of the PtdIns(3,4,5)P_3 levels was performed by calculating the average fluorescence intensity of separated regions of the cell normalised to the cell-region pixel-area and the laser power (fluorescence units, FU) in a section near the equatorial plane. The pixels corresponding to the PM, the cytoplasm and the nucleus were analysed separately by image segmentation. The ratio of the normalised intensity at the PM to that at the cytoplasm (PM/Cyt

ratio) was used to quantify differences in the translocation level of the GRP1^{PH} construct between cells of the same line. To facilitate the comparison, the PM/Cyt ratio of each cell was normalised to the average PM/Cyt obtained with a mCherry-6xGly-eGFP tandem in each cell line, used to quantify the level without translocation. The nucleus was excluded from the analysis by segmentation.

To quantify the accessible endogenous PtdSer content at the inner leaflet of the PM we used the discoidin-like C2 domain of bovine Lactadherin (LactC2) as a genetically encoded fluorescent biosensor that binds accessible PtdSer with high selectivity [2]. Imaging, transfection and segmentation were performed analogously to GRP1^{PH} as above. Reliable quantification was achieved by co-expressing mCherry-LactC2 and free-diffusing eGFP at a constant ratio by means of a self-cleaving P2A peptide (mCherry-LactC2-P2A-eGFP plasmid) [6]. As free-diffusing eGFP distributed homogeneously in the cytoplasm, its intensity was proportional to the total amount of mCherry-LactC2 in the cell. For every condition, the normalised average intensity of mCherry-LactC2 measured at the PM was plotted against the normalised average intensity of eGFP in the cytoplasm for a large population of cells and fit to a straight line. The slope of this linear regression allowed reliable quantification of relative PtdSer content at the PM in different conditions (Fig. S8).

Quantification of the Dimerisation Efficiency (E_D). The dimerisation efficiency, E_D, was calculated on a per cell basis for more than 30 cells per condition. All data was analysed following the methodology developed in [7]. The fluorescent decay from cells transfected with eGFP-PDK1 or eGFP-PH^{PDK1} was majorly monoexponential with a small contribution from a second component due to eGFP photophysics[8,9] and possibly, residual autofluorescence. The lifetimes $\tau_{GFP}^{(1)}$ and $\tau_{GFP}^{(2)}$ and the contribution of each component $f_{GFP}^{(1)}$ and $f_{GFP}^{(2)}$ to the overall fluorescent emission are dependent on the molecular environment and fixation methods. Donor-only samples were first studied to characterise their fluorescent decay in our specific experimental conditions. Spatially invariant lifetime analysis of all cells transfected with donor-only species yielded $\tau_{GFP}^{(1)}$=2.21 ±0.01 ns and $\tau_{GFP}^{(2)}$=1.09 ±0.7 ns for SKBR3 cells and 2.16 ±0.01 and 0.77 ±0.05 ns for NIH3T3 cells. The contribution of the shortest lifetime $f_{GFP}^{(2)}$ was 0.10 ±0.02 (N= 75 cells) for eGFP-PDK1 and eGFP-PH^{PDK1} irrespective of the cell line. No difference between mutants was found within the experimental uncertainty.

A spatially invariant analysis of the lifetime was performed on all images corresponding to a FRET pair (WT, mutant) and experimental condition (resting, Growth Factor -stimulated or PI3K -inhibited). It was assumed that only one molecular conformation for the interaction was

possible (or, if they were more, they were indistinguishable within the experimental uncertainty). The fluorescent decays could be satisfactorily fit to a double exponential model with randomly distributed residuals (Fig. S4 A-B). The donor long lifetime of donor and acceptor co-transfected samples was similar to the long-lifetime of donor-only samples, $\tau_{GFP}^{(1)}$, and was thus, identified as non-FRET donor[7]. The short lifetime was smaller than the short lifetime of donor-only samples τ_{FRET}^{eff}, (0.87 ±0.03 ns for SKBR3 cells and 0.72 ±0.03 ns for NIH3T3 cells) and its corresponding weight, f_D^{eff}, was higher. This component was, thus, identified as a mixture of donors involved in FRET and unperturbed donors relaxing with the fast-decaying component that had been previously identified in the absence of an acceptor[7]. In analogy to the simpler case of a monoexponentially decaying donor undergoing FRET, we termed this component an effective FRET component.

$$I(t) = \left(1 - f_D^{eff}\right) \cdot e^{-t/\tau_{GFP}^{(1)}} + f_D^{eff} \cdot e^{-t/\tau_{FRET}^{eff}} \qquad \text{(S1)}$$

The f_D^{eff} for the PDK1-PDK1 interaction was consistently different between mutants as well as before and after stimulation (Fig. S4C-D and S4A), confirming that the difference in FRET that we measured was due to a cellular response induced by the growth factor and not to random donor-acceptor encounters within the cell. Moreover, we experimentally ruled out non-specific FRET since the f_D^{eff} in the cytoplasm of cells that had been co-transfected with eGFP-PH^{PDK1} and free-diffusing mRFP was at the lower detection limit except for cells with the highest concentration of acceptor. In all cases it was below that of cells that had been co-transfected with eGFP-PH^{PDK1} and mRFP-PH^{PDK1}.

For a low affinity molecular interaction, as is PDK1-PDK1 homodimerisation[7], the slope of the linear regression of the fraction of bound donor as a function of the total acceptor concentration is roughly proportional to the affinity of the interaction[7] (Fig. S4D). The total acceptor concentration, A_T, is proportional to the fluorescence intensity of the acceptor. Therefore, under the low affinity assumption, the slope of the regression of f_D^{eff} as a function of acceptor fluorescence provides an estimate of the probability of PDK1 homodimerisation. In the absence of acceptor, no FRET should occur and thus the intercept A_T=0 is precisely the contribution of the fast-decaying component of eGFP, which had been also determined independently from donor-only samples ($f_{GFP}^{(2)}$) (Fig. S4D).

However, the quantification of its uncertainty is challenging due to cell-to-cell variability and the fact that the linear regression is only an approximation to complex equilibrium behaviour. Consequently, rather than the linear regression, we used the mean of the distribution of f_D^{eff} after correcting for the total intensity of the acceptor for every cell as an estimate of the probability of PDK1 homodimerisation, and its standard deviation as its uncertainty (Fig. S4E). This was, in turn, a measurement of the dimerisation efficiency (E_D) of PDK1.

$$E_D = \overline{\left(\frac{f_D^{eff} - f_{GFP}^{(2)}}{A_T} \right)}$$

PDK1-Akt/PKB interaction was quantified in an analogous fashion after imaging the FRET between eGFP-PDK1 and mCherry-Akt/PKB.

All data concerning TCSPC fitting, image registering, and segmentation was analysed using in-house developed software written in Matlab (available at request). TCSPC fitting algorithms performed iterative-reconvolution with the experimentally characterised IRF and accounted for low photon-count time-bins at the tail of the decay. The FLIMfit software tool developed at Imperial College London and TRI2 (Paul R. Barber, University of Oxford) were occasionally used to render FLIM images.

Segmentation of FRET-FLIM images. The E_D was first quantified for the whole cell. The image of every cell was afterwards segmented into plasma membrane, cytoplasm and nucleus based on the confocal intensity images (Fig. S4F) as explained above. The lifetime data for the cellular regions was fitted to a double exponential model fixing the long lifetime obtained from the spatially invariant analysis above. This way the E_D for the cellular regions was obtained.

Molecular modelling. All experiments were performed with 1W1D [10] structure retrieved from the Protein Data Bank (PDB). This structure of the PH domain was used because of its excellent resolution (1.5Å), and the presence of inositol-(1,3,4,5)-tetrakis phosphate (Ins(1,3,4,5)P_4) bound to the protein along with a glycerol molecule, suggesting binding sites. The N-terminal part of this structure corresponds to the linker between the PH domain and the kinase part (absent here). Accelrys Discovery Studio 3.1 was used to prepare the protein. Ligand ions, water molecules were removed manually and the resulting file subjected to the 'Clean Protein' module which allowed completion of all missing atoms or residues automatically. In this case, missing atoms were on the extremities and completed for the sake of coherence. The serine residue at position 410 was phosphorylated manually. After verifying the modifications and selecting alternate residues in order to minimise any steric hindrance, the protein was submitted to a

cycle of minimisations, first with a Steepest Descent (SD) then a Conjugate Gradients algorithm with constraints on the backbone. Glycerol (GLY) and Ins(1,3,4,5)P_4 were bound in the crystal at different sites which are biologically relevant[11]. During all the minimisation phases, both small molecules were considered as ligands and conserved then saved in different files before simulations. In order to check the viability of the phosphorylated protein, the obtained file was submitted to an equilibration simulation by molecular dynamics using GROMACS 5.0 package and the gromos 54a7 forcefield[12]. Protein was solvated with explicit water molecules and a 150 mM concentration of salt. The system was equilibrated in the constant-NVT (number of particles, volume and temperature) ensemble for 2 ns then in the constant-NPT (number of particles, pressure and temperature) ensemble for the same duration. For equilibrium purposes, positional restrains were applied on the protein backbone. Temperature and pressure were maintained at values of 300 K and 1 bar respectively. Since nothing is known about the phosphorylated PH domain, this calculation was performed to check its stability over 50 ns, asserting the possibility to use the 1W1D conformation, and the low RMSD obtained proved this assessment. During the rest of the simulations presented here, this conformation was kept rigid.

PtdSer was prepared after extraction from the PDB 3KAA structure, from which caproyl esters were removed[13]. PtdIns(3,4,5)P_3 was prepared from the Ins(1,3,4,5)P_4 molecule by adding a glycerol to the phosphate at position 1. Hydrogen atoms were added to all ligands, which were minimised briefly (SD algorithm) since all torsions were allowed during simulations. Vina 1.1.2 was used to dock small molecules, with full flexibility, while the receptor protein was kept rigid, as stated above. AutoDock Tools 1.5.6 were used to prepare the pdbqt files corresponding to the ligands and the receptor. Note that the fatty acid chains that should be present on both ligands were not considered here for the sake of simplification. Docking a ligand with two long carbon chains would not give accurate results in terms of geometry while lengthening considerably the calculation time. Once the ligand docked onto PDK1 surface, one has to view the fatty acid chains as perpendicular to the protein, embedded into a bilayer cellular membrane.

Three series of docking were obtained on the 'GLY site', in the 'inositol site' (site dockings) and on the whole surface of the receptor (blind docking) to assess any determinism. The respective boxes were used to define the docking site, taking into account the bound surface cavities with a 5Å margin in the first two cases, encompassing the whole receptor for the latter. This margin was reduced where the 'GLY site' and the 'inositol site' intersected to avoid encompassing the neighbouring cavity. For each docking site, five experiments were performed and analysed visually. The best poses (out of the maximum 20 obtained), those with the best

energy were considered when they were identical over five simulations, leading to pose convergence. The following poses were generally convergent over the five experiments, but with slight energy differences. The 'GLY site' proved to be irrelevant in our experiments. Since a reasonable margin was used, all solutions were bound around the cavity previously occupied by the glycerol molecule, not inside it as determined in the original crystal. Even glycerol was not found preferentially in its native conformation, probably meaning its presence was a crystallisation artefact. The 'inositol site' allowed binding of the inositol "ligands" with conformations nearly identical to the one observed in the crystal structure (RMSD<1Å). PtdSer also bound to this site, not exactly in the cavity but slightly on one side, occupying the site previously held by one of the inositol phosphates, and forbidding any more docking into this region.

When blind docked, the "ligands" showed preferences, of the utmost importance. Glycerol did not bind into the inositol or in the 'GLY site', probably confirming its lack of relevance as a ligand in the native crystal. It did not show any selectivity but was bound in nearly all cavities of the protein surface. The Ins(1,3,4,5)P_4 "ligand" bound the 'inositol site' albeit in a conformation slightly different from the crystal, while interacting with the known protein residues. On the contrary PtdIns(3,4,5)P_3 and PtdSer specifically bound to the 'inositol site' with a high selectivity. When docked this way, binding of the inositols simultaneously with PtdSer was thus impossible, which implied a competition for the same binding site between these "ligands". The energy of the docked ligands could be used to discriminate them, but considering the structural differences, and the small number of molecules this would seem abusive and was not the purpose of this study. None of the "ligands" specifically docked into the 'GLY site' in this experiment. One should note that the blind docking provided qualitative results about a ligand tendency to bind a specific area of the receptor.

In order to propose dimer formation, protein-protein docking was performed with the Patchdock server available on the Internet [14], using the automated procedure with no restrictions or preferences. Three PDK1 PH-domain structures were used in order to obtain complexes, where the proteins were rigid during the whole process. The first one was the one described above, that was used for docking small molecules without ligands in the active sites suggested in the crystal. The second one incorporated PtdIns(3,4,5)P_3 in the 'inositol site', as obtained by site docking (note that the pose was nearly identical to the crystal). The last one showed the PtdSer and was obtained by docking as described above. These three structures were combined with Patchdock in order to obtain all six possibilities and simulate dimerisation of PDK1 PH domain with or without ligands. Among the obtained solutions, the 10 best ones

were saved and examined visually, while Patchdock scoring was used to discriminate these. Three dimers were found with the highest scores among the 6 series, and compared by superimposition in order to identify these precisely with respect to the ‘inositol sites’ occupation. This means that one complex was found four times with different ligands. The presence of ligands in the ‘inositol site’ and a possible association with a membrane were checked to detect any structure hindrance forbidding the ligands to bind the lipid bilayer. This way, C1 and C2 complexes were recognised as able to bind a membrane through its ligand(s) in the ‘inositol site(s)’. On the contrary C3 displayed steric hindrance forbidding an access of PtdIns(3,4,5)P_3 to a lipid bilayer. The angles measured between the PH domain in the dimers were obtained through the linker’s direction after docking. This provided information about the proximity between the respective kinase domains in the obtained dimer.

Supplementary figures

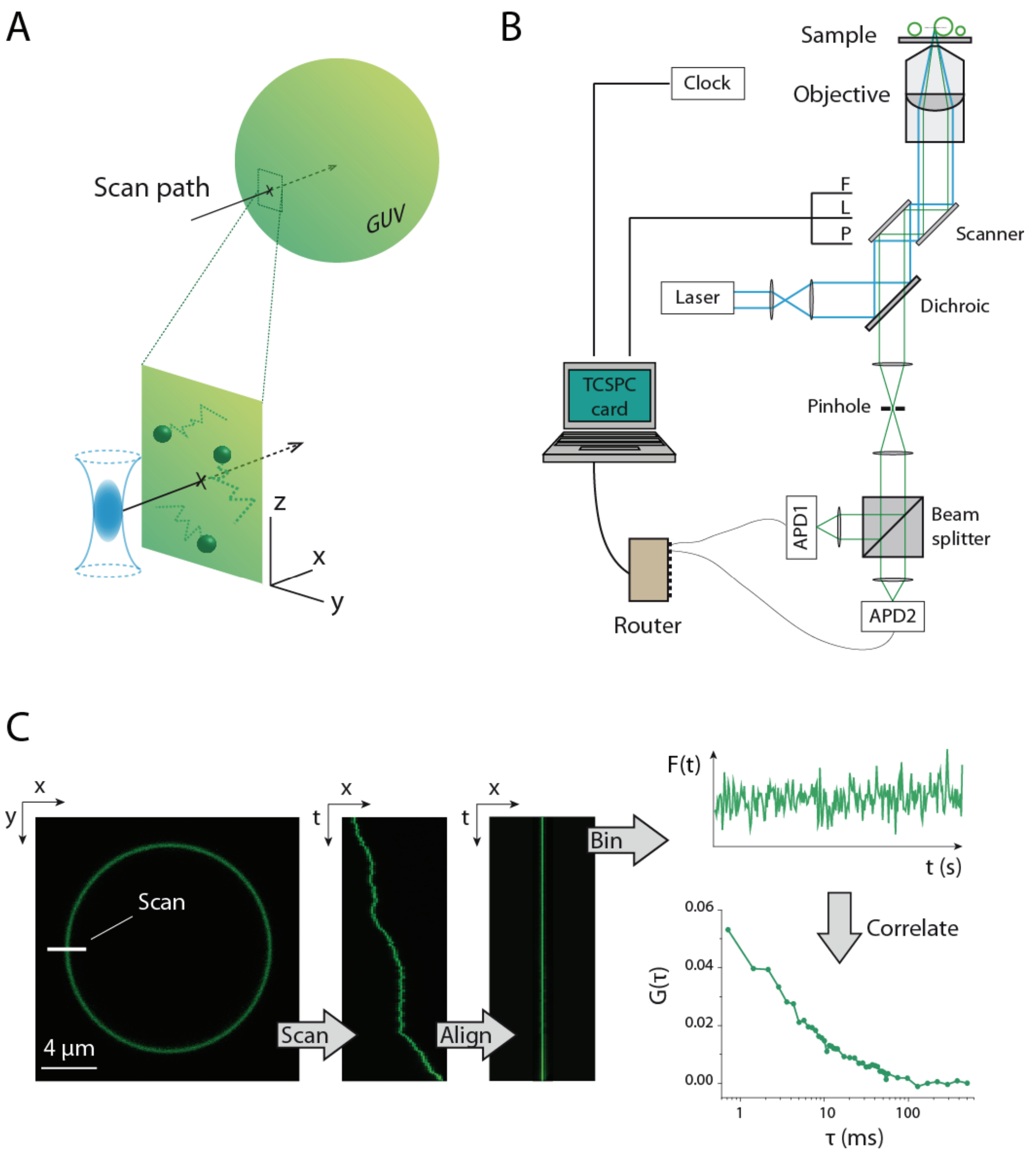

Fig. S1: Experimental implementation of scanning FCS. (A). Scanning FCS in GUVs was performed along a path perpendicular to the membrane plane (yz) and to the optical axis (z). **(B)** Instrumental configuration for point and scanning FCS, described in detail in the Methods section of the main text. F, L and P refer to the frame, line and pixel signals sent by the microscope to the TCSPC card in order to track the beam position at the sample. **(C)** The GUV was placed near its equatorial plane and a single line was scanned for 5 min at 1400 Hz. Due to the GUV drift the position of the membrane in the scanned line was shifted during the measurement. Rectification of the membrane position in time was done by alignment of the fluorescence maxima in each line. Spatial binning of the pixels corresponding to the GUV membrane allowed obtaining the fluorescence fluctuation time trace, which was used to calculate the autocorrelation function (ACF). The maximal temporal resolution of this ACF was limited by the scanning frequency.

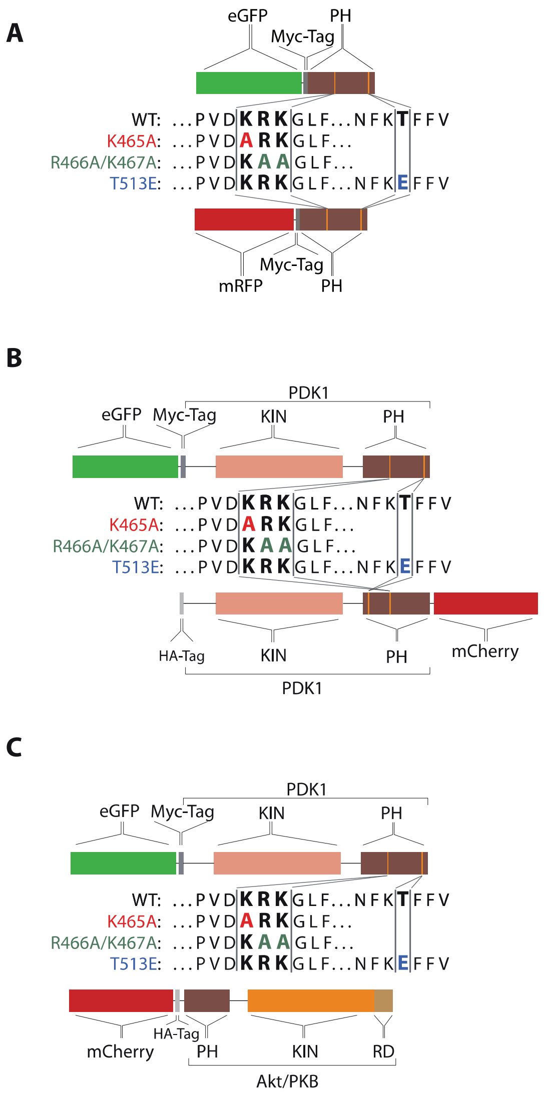

Fig. S2. Fluorescently-tagged PDK1, PH^{PDK1} and Akt/PKB constructs

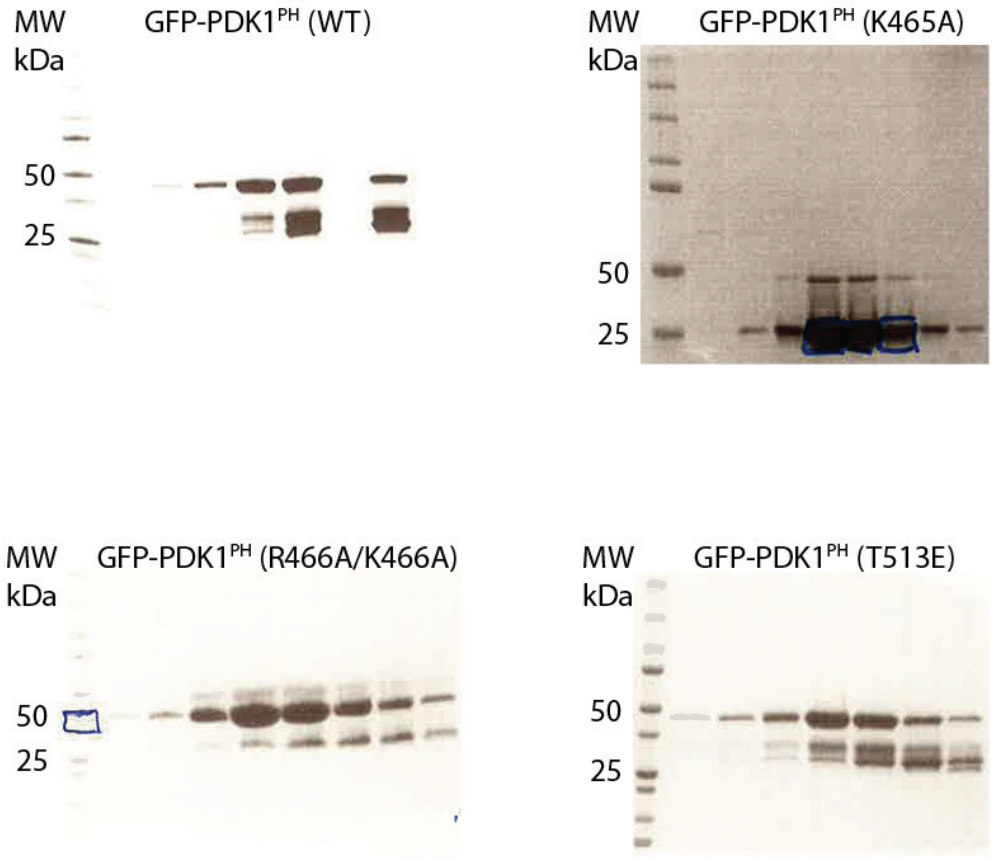

Fig. S3. Fractions of GFP-PH^{PDK1} stained with Coomassie following SDS-PAGE. All GFP-PH^{PDK1}fractions ran at the same expected size (≈48 kDa). The fractions with visible bands were pooled and concentrated. Cleavage of GFP-PDK1^{PH} can be observed (the two bands roughly at the size of the free protein EGFP and PDK1^{PH}), in all samples. This affected the quantification of the protein concentration, but did not affect the sFCS and protein-lipid overlay measurements since every mutant showed a different behaviour

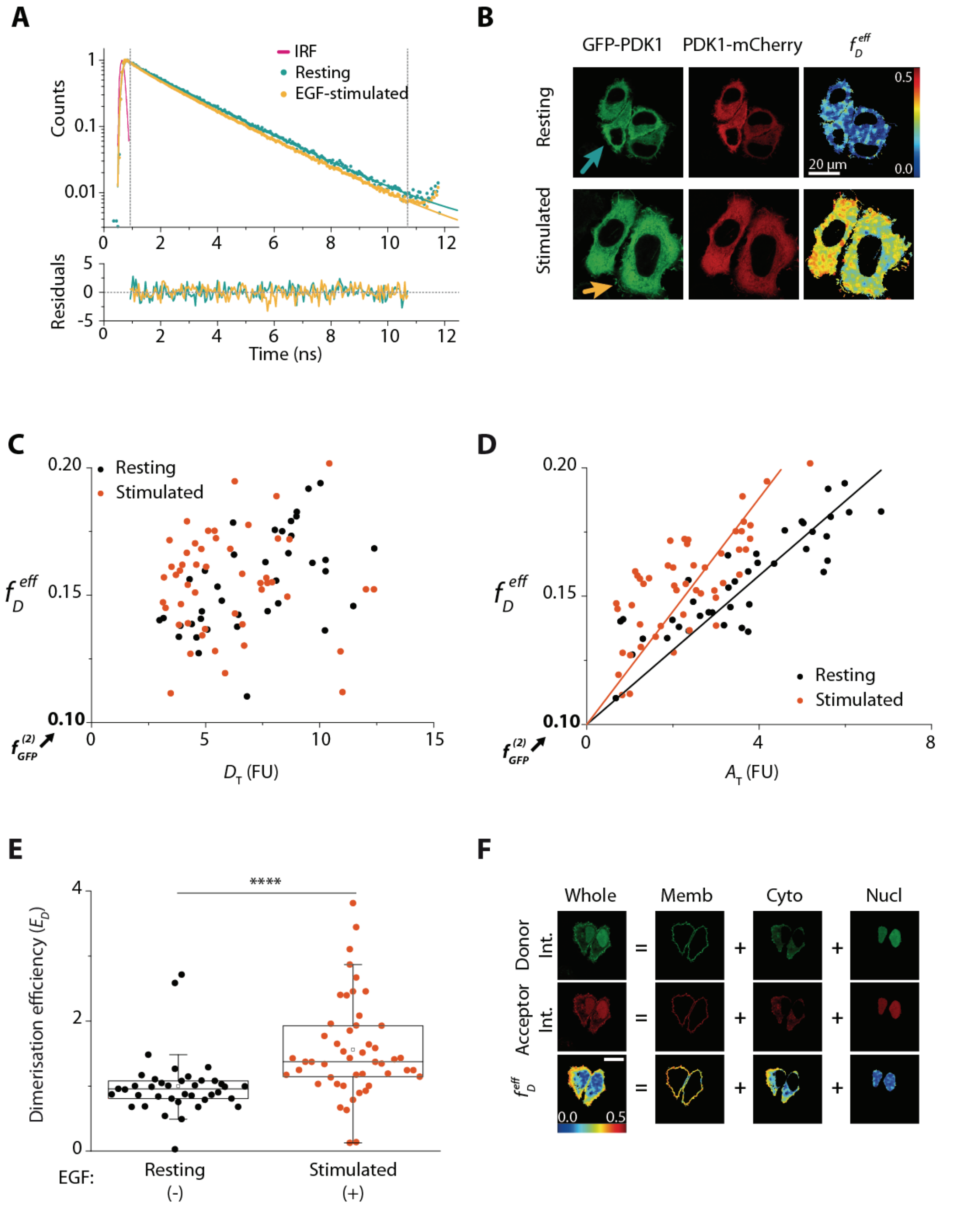

Fig. S4. Quantification of the binding efficiency of two proteins using Time-Resolved FRET (FRET-FLIM). (A-B) Cells were transfected with proteins tagged to a fluorescent donor or acceptor. The effective fraction of donor undergoing FRET, f_D^{eff}, was calculated fitting the fluorescence decay to a biexponential model keeping the two lifetimes fixed to those obtained by spatially invariant analysis. **(A)** Representative decays of cells in resting (blue arrow) and growth factor stimulated conditions (yellow arrow): cell in resting conditions: f_D^{eff} = 0.13, χ^2_r= 1.02; stimulated cell: f_D^{eff} = 0.26, χ^2_r= 1.06. **(C-D)** The fraction of interacting donor depends on the donor and acceptor concentration expressed in the cell (each dot is one cell), measured as intensity normalised to the cell pixel-area and the laser power (FU, Fluorescence Units). The f_D was found to be consistently higher after stimulation, ruling out that FRET is due to random-acceptor encounters. **(D)** Linear regression of f_D^{eff} as a function of the acceptor concentration for different physiological conditions. **(E)** Binding efficiency for the donor-tagged protein quantified from (D) for resting versus EGF-stimulated cells under the assumption of a low affinity interaction. **(F)** The image of every cell was segmented into plasma membrane, cytoplasm and nucleus based on the confocal intensity images and the lifetime was calculated for every region.

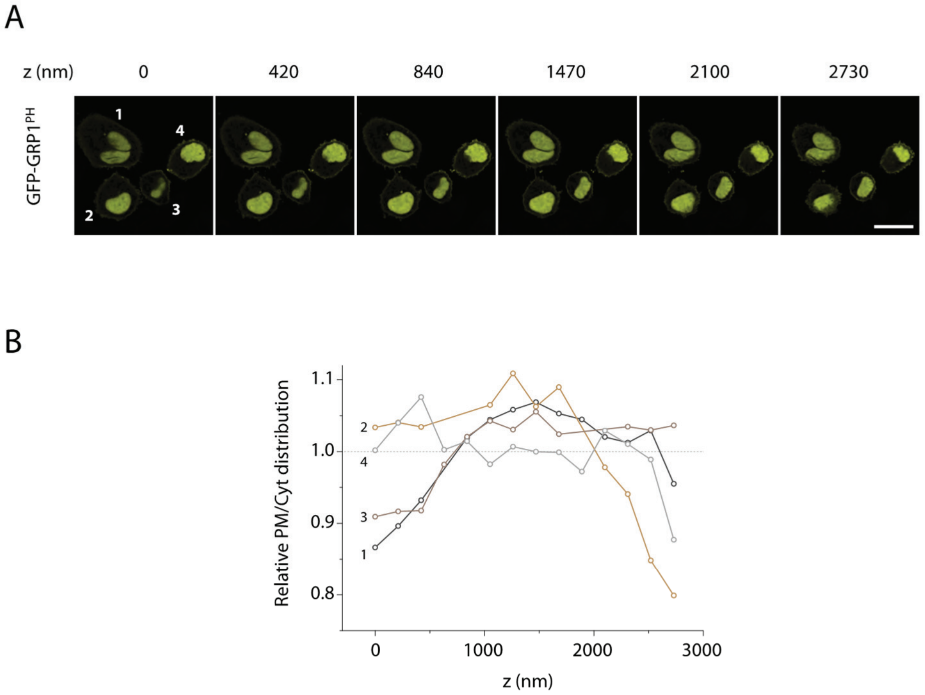

Fig. S5. Calculation of the plasma membrane to cytoplasm partition coefficient. The calculation of PM/Cyt intensity ratio is independent of the axial plane within roughly ±1 µm the equatorial plane of the cell. **(A)** Representative z-stack of SKBR3 cells expressing GFP-PH^{PDK1}. **(B)** The PM to cytoplasm ratio of the intensity of GFP-PH^{PDK1} was calculated for each axial plane and normalised to the average of each cell. The PM/Cyt intensity ratio was found constant within roughly one micron at each side the equatorial section. Cells were thus imaged in this region to avoid variability. Scale bar: 20 µm.

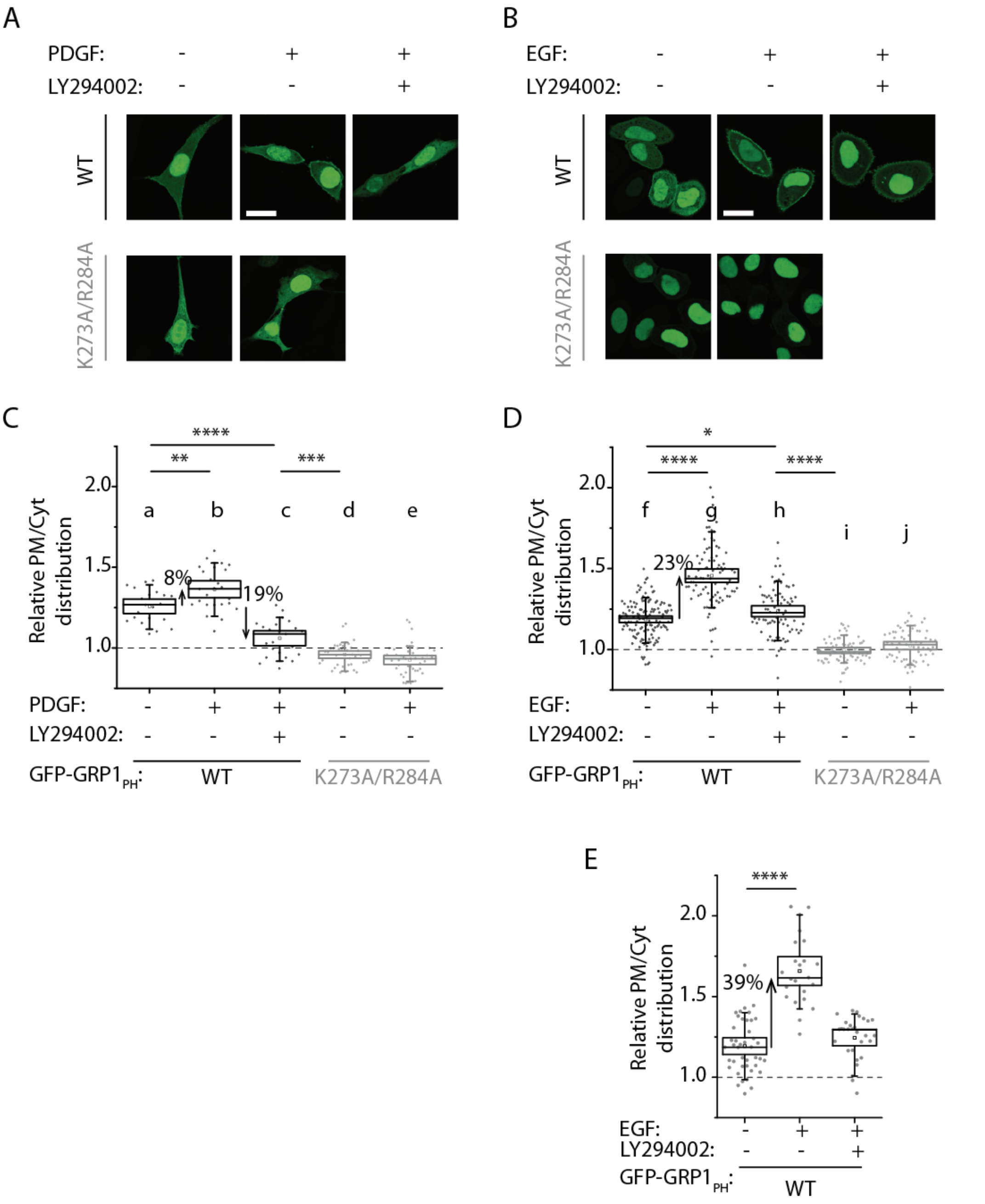

Fig. S6. Quantification of PtdIns(3,4,5)P_3 levels in intact NIH3T3 and SKBR3 cells using the high-selectivity PH domain probe GFP-GRP1^{PH}. Left: NIH3T3 cells; Right: SKBR3 cells. All experiments were performed in resting conditions (-), growth-factor stimulated (+) and treated with PI3K inhibitor LY294002 prior to stimulation. **(A-B)** Representative images of NIH3T3 (left) and SKBR3 (right) cells cotransfected with GFP-GRP1^{PH}. No association of the non-PtdIns(3,4,5)P_3-binding mutant (K273A/R284A) of GFP-GRP1^{PH} to the PM could be observed. **(C-D)** The amount of GRP1^{PH} accessible to PtdIns(3,4,5)P_3 at the PM upon stimulation of the PI3K pathway is higher for SKBR3 cells (right) than for NIH3T3 cells (left). When PI3K is inhibited prior to stimulation the amount of PtdIns(3,4,5)P_3 at the PM falls below the basal levels in NIH3T3 cells (C), but stays at basal level in SKBR3 (D). The dashed line indicates the reference level for a control cytoplasmic protein imaged with a 68 nm pixel size (Table 1). **(E)** Non-starved SKBR3 cells transfected with GFP-GRP1^{PH} were fixed and imaged in resting conditions, EGF-stimulated and treated with 50 µM LY294002 prior to stimulation to prevent PtdIns(3,4,5)P_3 generation by PI3K. The distribution of GFP-GRP1^{PH} at the PM relative to the cytoplasm is analogous to starved cells for the three conditions. This result confirms that the lack of decrease of the PtdIns(3,4,5)P_3 levels below basal in SKBR3 cells when PI3K is inhibited is not due to starvation (C). The absolute PtdIns(3,4,5)P_3 levels of the starved and non-starved cells are different. Scale bar: 20 µm. NIH3T3: N>20. SKBR3: N>30. Box: 2xSEM; Whiskers: 80% population. Mann-Whitney test $^{*}p<0.05$. Three independent experiments.

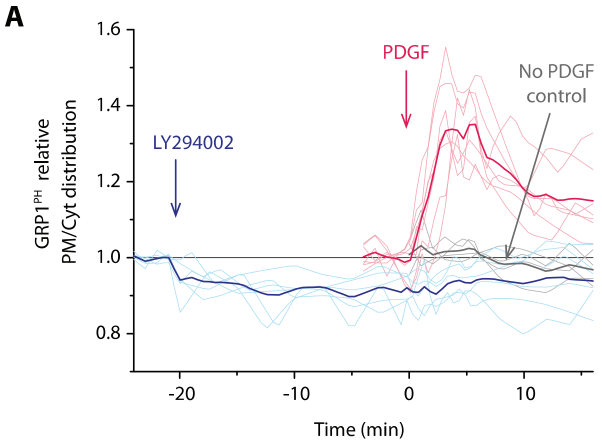

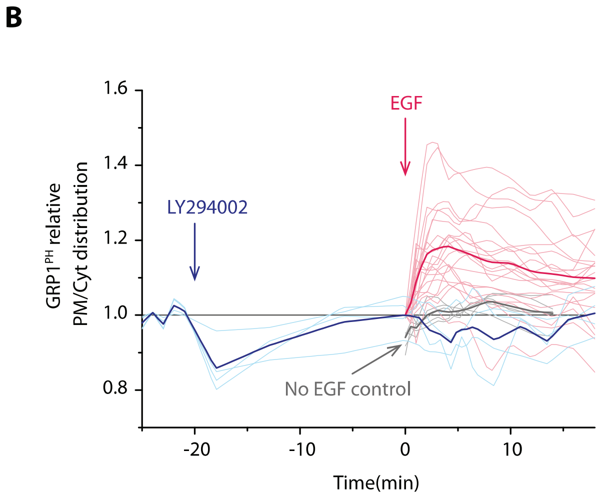

Fig. S7: Time course of PtdIns(3,4,5)P_3 levels at the plasma membrane in live NIH3T3 and SKBR3 cells using GFP-GRP1^{PH}. PtdIns(3,4,5)P_3 at the PM increases upon stimulation and is reduced below basal level after PI3K inhibition in live NIH3T3 (A), but not in SKBR3 (B) cells confirming observations in fixed cells. **(A)** NIH3T3 and **(B)** SKBR3 cells were transfected with GFP-GRP1^{PH} and imaged live at physiological conditions for more than 30 min after growth factor stimulation and/or inhibition of the PI3K pathway. The graph shows the distribution of the PH domain of GRP1 at the PM relative to the cytoplasm. The pink curves are cells stimulated with PDGF (NIH3T3) or EGF (SKBR3) at time 0; the blue ones are cells that had been treated with LY294002 for 20 min prior to stimulation at time 0; the grey curves are cells that had not been stimulated nor treated with the inhibitor. Highlighted curves are the average of the dimmer curves (NIH3T3: N_{PDGF}= 7; N_{LY}= 6; N_{CTL}= 4; SKBR3: N_{EGF}= 21; N_{LY}= 4; N_{CTL}= 5). The temporal distribution of single cells confirms the ensemble observations in fixed cells, shown in the main text.

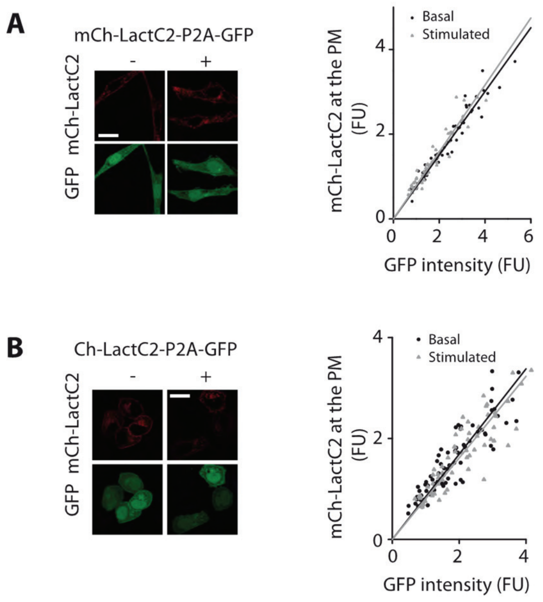

Fig. S8. Quantification of PtdSer levels at the PM of NIH3T3 and SKBR3 cells. (A) NIH3T3 and **(B)** SKBR3 cells were transfected with the multicistronic plasmid mCherry-LactC2-P2A-GFP, which translates mCherry-LactC2 and GFP separately. The images in the top row show the mCherry-LactC2 channel and the ones in the bottom row show the GFP channel. GFP serves as a transfection reporter and also allows LactC2 expression to be quantified due to the constant ratio of P2A translation. Cells on the right column were stimulated with PDGF (NIH3T3) or EGF (SKBR3) for 2 min prior to fixation. The average intensity of mCherry-LactC2 at the PM was plotted as a function of that of GFP over the whole cell for every cell and fitted to a linear model. Differences in PtdSer localisation at the PM were not observed before or after growth factor stimulation. Scale bar: 20 µm. N>30. Three independent experiments.

A

Conserved aminoacids in the T-loop of PKCγ and SGK1

PKCγ: 500- M **CKEN**VFPGT **TT** RT **FCGTP** D **Y** I**APE** IIAYQ -531
SGK1: 240- FGL**CKEN** IEHNS **TT** ST **FCGTP** E **Y** L**APE** VLH -271

B

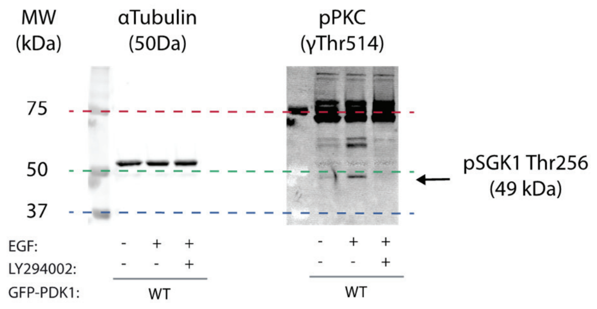

Fig. S9: Phospho-PKC(pan) (γThr 514) detects phosphorylation of endogenous SGK1 at its T-loop. (A) The activation segment (T-loop) of PKC and SGK1, located in their catalytic domain, is a highly conserved regulatory motif. The phosphorylation residues for PKCγ (Thr 514) and SGK1 (Thr 256) are in red. **(B)** The phospho-PKC antibody efficiently recognises the T-loop of several AGC kinases, including SGK1 and PDK1. Endogenous phosphorylated SGK1 in Fig. 5D was quantified at the 49 kDa band (see Methods).

Supplementary references

1 Masters, T. A. *et al.* Regulation of 3-Phosphoinositide-Dependent Protein Kinase 1 Activity by Homodimerization in Live Cells. *Sci. Signal.* **3**, ra78, doi:ra78

10.1126/scisignal.2000738 (2010).

2 Yeung, T. *et al.* Membrane phosphatidylserine regulates surface charge and protein localization. *Science* **319**, 210-213, doi:10.1126/science.1152066 (2008).

3 Najafov, A., Sommer, E. M., Axten, J. M., DeYoung, M. P. & Alessi, D. R. Characterization of GSK2334470, a novel and highly specific inhibitor of PDK1. *Biochem. J.* **433**, 357-369, doi:10.1042/bj20101732 (2011).

4 Collins, B. J., Deak, M., Murray-Tait, V., Storey, K. G. & Alessi, D. R. In vivo role of the phosphate groove of PDK1 defined by knockin mutation. *Journal of Cell Science* **118**, 5023-5034, doi:10.1242/jcs.02617 (2005).

5 Gray, A., Van der Kaay, J. & Downes, C. P. The pleckstrin homology domains of protein kinase B and GRP1 (general receptor for phosphoinositides-1) are sensitive and selective probes for the cellular detection of phosphatidylinositol 3,4-bisphosphate and/or phosphatidylinositol 3,4,5-trisphosphate in vivo. *Biochem. J.* **344**, 929-936, doi:10.1042/0264-6021:3440929 (1999).

6 Szymczak, A. L. *et al.* Correction of multi-gene deficiency in vivo using a single 'self-cleaving' 2A peptide-based retroviral vector. *Nat. Biotechnol.* **22**, 589-594, doi:10.1038/nbt957 (2004).

7 de Las Heras-Martinez, G., Andrieu, J., Larijani, B. & Requejo-Isidro, J. Quantifying intracellular equilibrium dissociation constants using single-channel time-resolved FRET. *J. Biophotonics* **11**, doi:10.1002/jbio.201600272 (2018).

8 Visser, A. *et al.* Time-resolved FRET fluorescence spectroscopy of visible fluorescent protein pairs. *Eur. Biophys. J. Biophys. Lett.* **39**, 241-253, doi:10.1007/s00249-009-0528-8 (2010).

9 Heikal, A. A., Hess, S. T. & Webb, W. W. Multiphoton molecular spectroscopy and excited-state dynamics of enhanced green fluorescent protein (EGFP): acid-base specificity. *Chem. Phys.* **274**, 37-55, doi:10.1016/s0301-0104(01)00486-4 (2001).

10 Komander, D. *et al.* Structural insights into the regulation of PDK1 by phosphoinositides and inositol phosphates. *Embo J.* **23**, 3918-3928, doi:10.1038/sj.emboj.7600379 (2004).

11 Lucas, N. & Cho, W. Phosphatidylserine Binding Is Essential for Plasma Membrane Recruitment and Signaling Function of 3-Phosphoinositide-dependent Kinase-1. *J. Biol. Chem.* **286**, 41265-41272 (2011).

12 Van Der Spoel, D. *et al.* GROMACS: fast, flexible, and free. *Journal of computational chemistry* **26**, 1701-1718, doi:10.1002/jcc.20291 (2005).

13 DeKruyff, R. H. *et al.* T cell/transmembrane, Ig, and mucin-3 allelic variants differentially recognize phosphatidylserine and mediate phagocytosis of apoptotic cells. *Journal of Immunology* **184**, 1918-1930, doi:10.4049/jimmunol.0903059 (2010).

14 Schneidman-Duhovny, D., Inbar, Y., Nussinov, R. & Wolfson, H. J. PatchDock and SymmDock: servers for rigid and symmetric docking. *Nucleic Acids Research* **33**, W363-W367, doi:10.1093/nar/gki481 (2005).
